# Strong and corrosion-resistant 3D-printed steel by self-assembled core-shell nanoparticles

**DOI:** 10.1126/sciadv.aea5057

**Published:** 2026-06-10

**Authors:** Wenhua Wu, Yuxuan Zhao, Dong Qiu, Guofeng Zhang, Youyou Zhang, Yifan Zhao, Gang Sha, Mingxing Zhang, Hongbiao Dong, Hao Chen

**Affiliations:** ^1^ Key Laboratory for Advanced Materials of Ministry of Education, School of Materials Science and Engineering, Tsinghua University, Beijing 100084, P. R. China.; ^2^ MOE Key Laboratory of Materials Physics and Chemistry under Extraordinary Conditions, School of Physical Science and Technology, Northwestern Polytechnical University, Xi’an 710072, P. R. China.; ^3^ State Key Laboratory of Metallic Materials for Marine Equipment and Applications, Beijing 100084, P. R. China.; ^4^ Centre for Additive Manufacturing, School of Engineering, RMIT University, Melbourne, VIC 3001, Australia.; ^5^ School of Materials Science and Engineering/Herbert Gleiter Institute of Nanoscience, Nanjing University of Science and Technology, Nanjing 210094, P. R. China.; ^6^ School of Mechanical and Mining Engineering, The University of Queensland, Brisbane, QLD 4072, Australia.; ^7^ Department of Engineering, University of Leicester, Leicester LE1 7RH, UK.; ^8^ Institute for Materials Research, Tohoku University, 2-1-1 Katahira, Aoba-ku, Sendai 980-8577, Japan.

## Abstract

Metal additive manufacturing often produces coarse columnar grains and elemental segregation, resulting in anisotropic mechanical properties and degraded corrosion resistance. We present a powder blending strategy using multicomponent carbides (MCCs) to overcome these limitations in 316L stainless steel. Upon dissolution, MCCs drive the self-assembly of uniformly distributed core-shell oxynitride-carbide nanoparticles, which sequester detrimental nitrogen/oxygen impurities and markedly refine austenite grain size from 43.9 to 2.1 micrometers. This unique microstructure control yields an excellent combination of strength and ductility. Crucially, the corrosion resistance is enhanced by suppressing chromium segregation via tungsten, niobium, and tantalum partitioning to the cell boundaries and facilitating the formation of a protective tungsten trioxide–rich passive film. This work establishes an instructive paradigm for metal additive manufacturing, demonstrating how MCCs’ introduction can tailor nanoprecipitations, grain structure, and alloy chemistry to simultaneously improve strength and corrosion resistance in structural alloys.

## Teaser

Multicomponent carbides refine grains and improve mechanical and corrosive properties of additively manufactured steels.

## INTRODUCTION

Laser additive manufacturing (LAM) has emerged as a transformative technology for fabricating complex metal components through layer-by-layer deposition (*1*, *2*). However, the rapid solidification conditions inherent to LAM, characterized by high cooling rates and steep thermal gradients, promote epitaxial grain growth, often leading to strongly textured and columnar grains in alloys such as 316L stainless steel, IN718, and TC4 alloys (*3*–*8*). These anisotropic structures not only degrade mechanical performance but also increase susceptibility to solidification cracking, posing a critical challenge to their structural applications (*9*–*11*).

Current strategies for inducing columnar-to-equiaxed transition (CET) in LAM fall into two categories: physical and chemical approaches (*12*–*18*). Physical methods such as ultrasonic vibration can effectively fragment dendrites in titanium and nickel alloys (*12*) but are incompatible with laser powder bed fusion (LPBF) due to powder bed disruption (*13*). Although using cold rolling can also effectively refine austenite grain size and improve mechanical properties, the increased dislocation density deteriorates the corrosion resistance (*14*). Chemical approaches using heterogeneous nucleants (TiC, NbC, and SiC) show greater promise for LPBF, with efficacy governed by four critical factors: interfacial energy, crystallographic matching, chemical stability, and melt wettability (*17*). Beyond promoting heterogeneous nucleation, these carbides can pin grain boundaries, facilitating CET and refining grain structure (*19*–*21*). However, conventional carbides exhibit inherent limitations under LPBF’s extreme thermochemical conditions (*22*–*25*). A major challenge is the tendency of carbides to segregate at grain boundaries, where agglomeration worsens with increasing carbide content (*26*). In addition, carbon dissolution could lead to chromium depletion, consequently degrading corrosion resistance (*27*). A critical but often neglected factor is oxygen either from the printing atmosphere or adsorbed on powder surfaces, which can chemically modify nucleant particles. For example, TiC refiners in 316L stainless steel may first melt and then undergo preferential oxidation, forming inert TiO_2_ phase before solidification (*23*). This reaction reduces crystallographic compatibility, accelerates particle coarsening to micro sizes, and ultimately diminishes grain refinement efficiency.

To overcome these limitations, especially the detrimental effects of N/O impurities, we propose a powder blending strategy using (TiWNbTa)C multicomponent carbides (MCCs) instead of the conventional carbides to refine the microstructure and enhance the performance of LPBF-fabricated 316L steel. Ti was firstly incorporated primarily as a potent getter for N/O impurities to form Ti(N,O) compounds, and then a multicomponent approach (W, Nb, and Ta) was used to maximize carbide stability at elevated temperature through high-entropy design principles and electronegativity synergies. This core-shell structure can suppress oxynitride coarsening, and doping TiC with Nb and W enhances heterogeneous nucleation efficiency by increasing adsorption energy and interface wettability, achieving a substantially higher active refiner particle density and more uniform distribution. The MCC reinforcement outperforms conventional strengthening phases in laser-additively manufactured 316L steels. Furthermore, the optimized chemical composition of MCCs inhibits Cr segregation and produces a protective WO_3_ passive film, improving the corrosion resistance of 316L-MCC steels. Our work thus provides a pathway to simultaneously control microstructure and enhance properties in LPBF-fabricated structural alloys.

## RESULTS

### Microstructural evolution in LPBF-fabricated steels

**Fig. 1.**
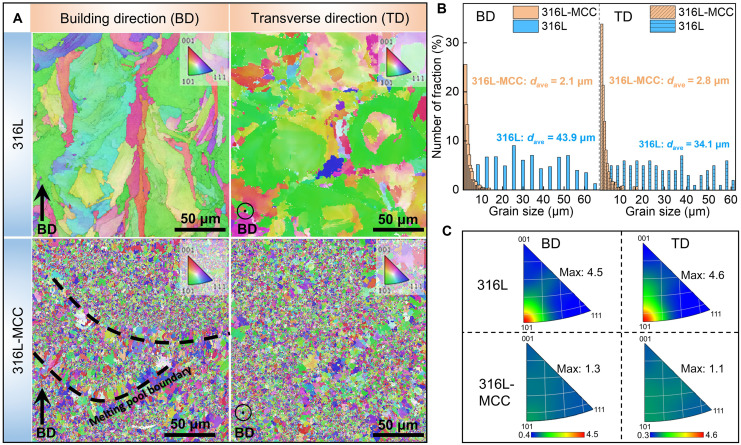
Crystallographic characteristics of LPBF-fabricated 316L and 316L-MCC samples. (**A**) Electron backscatter diffraction (EBSD) grain orientation maps along the BD and the TD. (**B**) The grain size distribution statistics of LPBF-fabricated 316L and 316L-MCC samples. (**C**) Corresponding inverse pole figures along different directions.

Figure 1 presents the crystallographic characteristics of LPBF-fabricated 316L and 316L-MCC steels, processed using optimized laser power of 210 W and scanning speed of 950 mm/s. The microstructure of the 316L sample exhibits coarse, columnar austenite grains elongated along the building direction (BD), presenting an epitaxial growth across successive layers, as shown in Fig. 1A. The length along the BD can reach >200 μm. In contrast, the addition of (TiWNbTa)C MCCs (4 wt %) yields a notable transition to an ultrafine, equiaxed grain structure with most grain sizes smaller than 10 μm. Notably, austenite grains at melt pool boundaries (delineated by dark dashed lines) are coarser than those within the melt pool interior, likely due to the higher thermal gradient at the boundary and Marangoni convection driving carbides to move away from the boundary. Besides, the 316L sample along transverse direction (TD) presents a typical cellular grain dominated by scanning rotation and hatching space, while this structure is completely eliminated and replaced by a mass of refined grains within the 316L-MCC sample. Quantitative analysis (Fig. 1B) reveals that the addition of MCCs refines the austenite grain size from 43.9 to 2.1 μm along BD direction and from 34.1 to 2.8 μm along TD direction. For comparison, we also added 1 wt % TiC (with the same carbon fraction) and 4 wt % TiC (with the same carbide fraction) into 316L steels, and austenite grains were respectively refined to 25.8 and 5.2 μm, as shown in fig. S1. This confirms that while TiC provides some grain refinement, it is less effective than MCC. According to crystallographic texture analysis (Fig. 1C), the LPBF-fabricated 316L steel presents a strong <1 0 1> texture along the BD, which is effectively suppressed by the addition of MCCs. These findings collectively demonstrate that the MCC introduction has superior ability to facilitate CET and grain refinement.

**Fig. 2.**
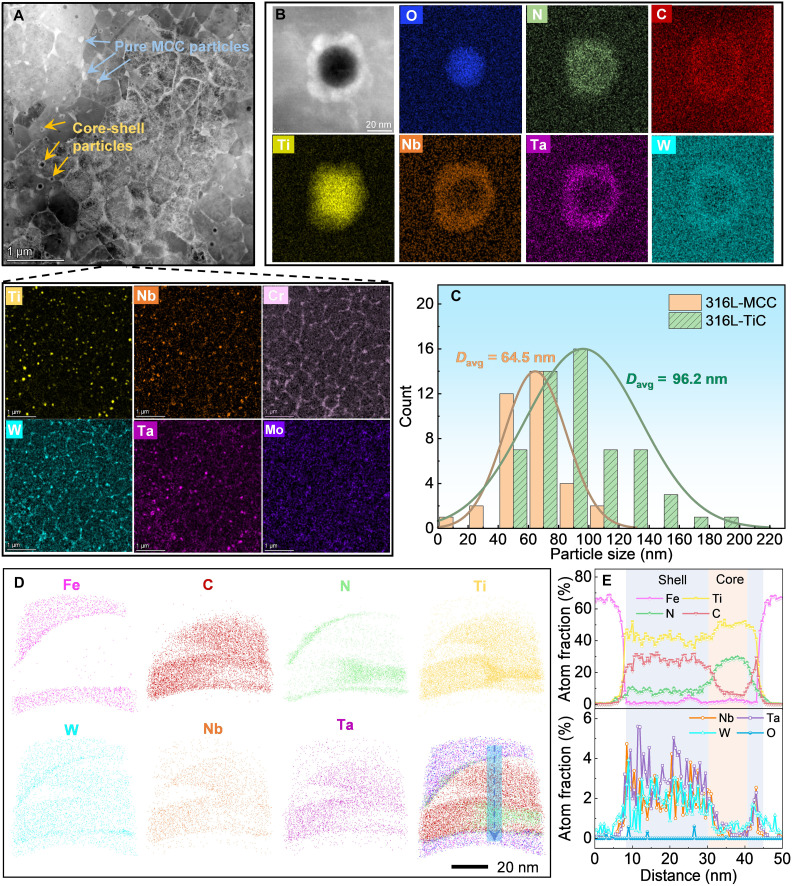
Microstructures and nanoscale element distribution of the LPBF-fabricated samples. (**A**) TEM and EDS images of the 316L-MCC sample, showing the distribution of nanoparticles and the segregation of Cr, Mo, Nb, Ta, and W along the cell boundaries. (**B**) TEM and EDS images of a nanoparticle showing the Ti(N,O) core and (Ti,Nb,Ta,W)C shell. (**C**) Particle size distribution in the 316L-MCC and 316L-TiC samples. (**D**) Reconstructed APT tips of Fe, C, N, Ti, W, Nb, and Ta atoms. (**E**) Concentration profiles across the austenite matrix and core-shell particle (the blue line in Fig. 2D).

The distribution and type of nanoparticles within the 316L-MCC sample are characterized using transmission electron microscopy (TEM), energy-dispersive x-ray spectroscopy (EDS), and three-dimensional atom probe tomography (3D-APT), as illustrated in Fig. 2. Figure 2A reveals typical cellular structures in the 316L-MCC sample. Elements such as Nb, W, Ta, Cr, and Mo are observed to segregate at the cell boundaries. Cr and Mo segregation is common at the cell boundaries of LPBF-fabricated 316L sample (*28*). This suggests that the MCC particles introduced into the 316L steel matrix may undergo partial or complete dissolution during the LPBF process, promoting the diffusion of W, Nb, and Ta elements to the cell boundaries. Unlike conventional LPBF-fabricated 316L, where Si is typically found in oxides (*5*), we observe that Si is incorporated into a fraction of the core-shell precipitates, with a pronounced enrichment at the shell regions (fig. S2). A high density of nanoparticles is uniformly dispersed throughout the 316L-MCC matrix. Many exhibit a core-shell structure (white core, black shell) and are primarily located within cell interiors. In contrast, pure nanoparticles with irregular shapes are predominantly found at cell boundaries, especially at the junction of different cells. The introduction of MCCs into 316L steels greatly decreases the fraction of low-angle grain boundary from 29.4 to 1.9%, accompanied by the slightly decrease of the kernel average misorientation (fig. S3). This indicates that the MCCs’ addition transformed the solidification behavior by promoting heterogeneous nucleation and equiaxed grain growth over epitaxial columnar growth, consequently suppressing the formation of subgranular structures. Meanwhile, the density of geometrically necessary dislocation increases from 1.73 × 10^14^ to 2.53 × 10^14^ m^−2^ (fig. S4).

The chemical composition of a core-shell nanoparticle is shown in Fig. 2B, where the core exhibits strong Ti, O, and N signals, while the shell comprises Ti, Nb, Ta, W, and C. TEM-EDS analysis (fig. S5) confirms that the white particles in Fig. 2A are MCCs consisting of Ti, Nb, Ta, W, C, and N. It is proposed that during rapid solidification, a portion of the Ti reacts with residual O and N to form Ti(N,O). Subsequently, MCCs either nucleate heterogeneously on Ti(N,O) surfaces, forming core-shell structures, or homogeneously nucleate in the matrix. For comparison, we analyzed nanoparticles in 316L–4 wt % TiC samples (fig. S6). Notably, the 316L-TiC sample primarily contains coarse Ti(C,N) particles, indicating that the dissolved TiC particles are chemically modulated by residual nitrogen. There are network-like carbide structures along the cell boundaries, which are formed by the eutectic reaction (*29*). Figure 2C shows statistical analysis of effective refiner particles (>10 nm). The 316L–4 wt % MCC sample exhibits a substantially smaller particle size (*D*_avg_ = 64.5 nm) compared to 316L–4 wt % TiC (*D*_avg_ = 96.2 nm). Two types of precipitates are identified in the 316L-MCC samples: pure MCCs and core-shell particles. Statistical analysis reveals that the core-shell particles, with an average size of 62.7 nm and a number density of 1.2 × 10^13^ m^−2^, are both more numerous and finer than the pure MCCs (74.5 nm and 4.3 × 10^12^ m^−2^). This indicates that the core-shell structure is the predominant precipitate morphology. The inhomogeneous distribution of detrimental N and O elements leads to variations in the core composition of the nanoparticles. As demonstrated in Fig. 2 (D and E), reconstructed APT images reveal a distinct nanoparticle with a core-shell structure. Quantitative analysis identifies the core as a titanium-rich nitride, while the shell is a carbon-rich (Ti, W, Nb, and Ta) carbonitride, with an atomic composition of ~30% C and <10% N. This observation confirms that the MCC shell effectively encapsulates the TiN core, ensuring a stable core-shell structure even when the core composition is influenced by local N and O concentration variation. An additional slice taken from a marked region (fig. S7), viewed from the top, also presents the core-shell structure of precipitate.

The 316L-MCC system incorporates multiple carbide-forming elements (Ti, W, Ta, and Nb). While Ti is preferentially consumed by N and O, the remaining elements (W, Ta, and Nb) bond with C to form carbides. These carbides adsorb onto Ti(N,O) surfaces, suppressing the coarsening of Ti(N,O) while using them as nucleation sites to increase the number density of MCCs. This synergistic mechanism explains the superior grain-refining efficiency of MCC compared to conventional TiC refiners.

### Mechanical property and corrosion resistance

**Fig. 3.**
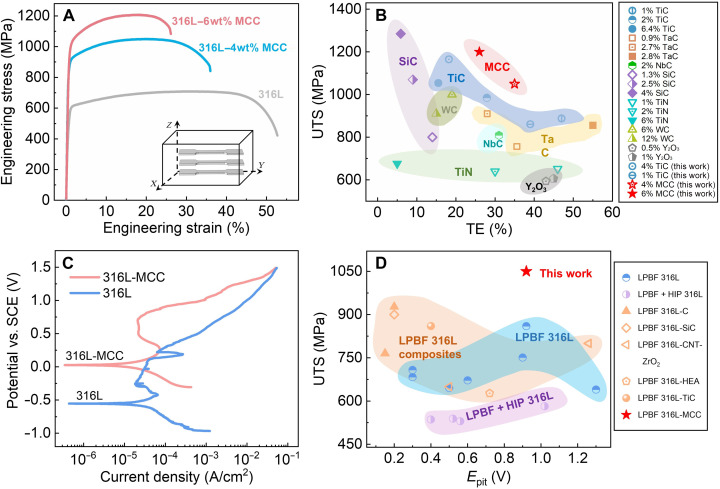
Mechanical property and corrosion resistance of the investigated steels. (**A**) Tensile stress-strain curves of LPBF-fabricated 316L and 316L-MCC samples. (**B**) Comparison of UTS and TE for the present 316L-MCC steels with other LPBF-fabricated 316L steel–based composites reported in the literature, including TiC (*29*, *40*, *41*), TaC (*42*), NbC (*43*), SiC (*22*, *44*), WC (*45*), TiN (*20*, *46*), and Y_2_O_3_ (*47*). (**C**) Potentiodynamic curves showing higher corrosion potential and a stable passive district with the addition of MCCs. SCE, saturated calomel electrode. (**D**) Comparison of UTS and pitting potential (*E*_pit_) for the present 316L–4 wt % MCC steel with other 316L variants, including standard LPBF (*48*–*51*), LPBF with hot isostatic pressure (HIP) (*52*, *53*), and other LPBF-fabricated 316L composites, including graphene (*54*), SiC (*55*), CNT-ZrO_2_ (*56*), FeCoNiCr high entropy alloy (*57*), and TiC (this work).

Figure 3A presents the tensile curves of 316L and 316L-MCC alloys. The LPBF-fabricated 316L steel exhibits yield strength (YS) and ultimate tensile strength (UTS) of 452 and 708 MPa, respectively, with 30.6% uniform elongation (UE) and 52.6% total elongation (TE). The 316L–4 wt % MCC composite demonstrates superior mechanical performance, achieving 836 MPa of YS and 1050 MPa of UTS. The YS has almost doubled while maintaining 20.0% UE and 36.0% TE. When the addition of MCCs increases to 6 wt %, the UTS reaches 1205 MPa with a TE of 25%, representing an excellent balance between strength and ductility. We also printed other 316L-based composites (316L-TiC and 316L-Cr_3_C_2_) using similar laser parameters, while their tensile performance is inferior to that of 316L-MCC composites (fig. S8A). Comparative analysis (Fig. 3B) reveals that the MCC reinforcement outperforms conventional strengthening phases (TiC, TaC, NbC, SiC, WC, TiN, and Y_2_O_3_) in laser-additively manufactured 316L composites. The effects of powder recycling time and build volume on mechanical properties are also evaluated to be minor (fig. S8B), confirming the robustness of 316L-MCC powders. To evaluate mechanical anisotropy, we compared tensile properties along the BD and the TD for both materials (fig. S8C). While both steels exhibit lower strength and ductility along the BD, the 316L-MCC steel shows a markedly smaller reduction in these properties. This indicates that the MCC addition mitigates anisotropy, a finding consistent with the more isotropic microstructure revealed in Fig. 1C.

Corrosion resistance is a critical performance index for austenitic stainless steel. As illustrated in Fig. 3C, the potentiodynamic polarization curves reveal that the addition of 4 wt % MCC particles markedly shifts the corrosion potential from −0.55 to 0.03 V, while the corrosion current density decreases by an order of magnitude, from 1.75 × 10^−5^ to 7.46 × 10^−6^ A/cm^2^, demonstrating a substantially reduced corrosion susceptibility with the addition of MCCs. Moreover, the passive film of the 316L sample is unstable, exhibiting breakdown and repassivation behavior. In comparison, 316L-MCC steel sample displays a clear passive district before pitting, where the current density remains nearly invariable as the polarization potential increases, indicating inactive corrosion with passivation and generating a very stable passive film. Figure 3D shows the comparison of UTS versus pitting potential (*E*_pit_) obtained in the current 316L–4 wt % MCC steels with previously reported LPBF-fabricated 316L–based materials. It is clear that 316L-MCC composites have the excellent combination of UTS and *E*_pit_, confirming that the addition of MCCs can simultaneously improve strength and corrosion resistance in AM-fabricated structural alloys.

Further insights were obtained through electrochemical impedance spectroscopy (EIS). The Nyquist plots (fig. S9A) exhibit a larger semicircular arc radius for the 316L-MCC sample, indicative of superior charge-transfer resistance. This observation is corroborated by the Bode plots (fig. S9B), where the 316L-MCC sample exhibits an impedance modulus of 2.97 × 10^5^ ohm·cm^2^, more than three times higher than that of unmodified 316L (9.17 × 10^4^ ohm·cm^2^). In addition, the phase angle of the 316L-MCC composite exceeds 80°, further confirming its enhanced passivation stability compared to conventional 316L steel. Collectively, the potentiodynamic polarization and EIS tests indicate that the incorporation of MCC particles enhances the corrosion resistance of 316L steel. Notably, we also compared the potentiodynamic polarization curves between 316L–4 wt % MCC and 316L–1 wt % TiC, as shown in fig. S9C. The 316L-MCC sample has a higher corrosion potential, and there is a stable passivation range, demonstrating superior corrosion resistance than that of 316L-TiC sample.

## DISCUSSION

### Precipitation behavior and grain refinement

**Fig. 4.**
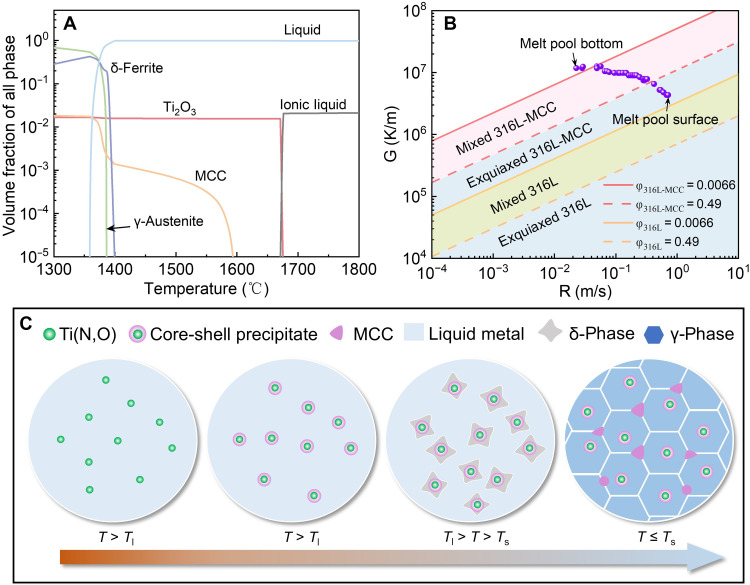
MCC formation and CET mechanisms of the 316L-MCC steels. (**A**) The equilibrium phase diagram of 316L-MCC sample calculated by Thermo-Calc. (**B**) Temperature gradient-solidification rate (G-R) map for 316L and 316L-MCC samples, and the purple symbols showing the simulated G and R within the melt pool by finite element simulation. (**C**) Schematic sketch showing the precipitation behavior of core-shell nanoparticles and its effect on the grain nucleation and growth during 316L-MCC steel cooling, where *T*_l_ and *T*_s_ are liquidus and solidus, respectively.

To elucidate the precipitation behavior of MCCs, we used Thermo-Calc software to calculate the equilibrium phase diagram of the 316L-MCC system, as illustrated in Fig. 4A. Notably, the nitrogen and oxygen contents in the sample were quantitatively measured to be 0.062 and 0.04 wt %, respectively. The thermodynamic calculations reveal that Ti_2_O_3_ precipitates as the primary phase at 1675°C, followed by the formation of the MCC phase at 1592°C. As demonstrated in fig. S10A, the MCC phase initially exists as Ti(N,C) in the liquid metal and subsequently transforms into (TiWNbTa)C carbide with limited nitrogen solubility in the mushy zone, which is consistent with the APT result in Fig. 2E. These two types of precipitates form sequentially before solidification, thereby easily forming a core-shell architecture.

To evaluate the grain refinement effect of MCCs on the solidification morphology, a Gäumann’s modified Hunt CET criterion (*30*) was implemented, as visualized in the G-R phase diagram (Fig. 4B). Demarcated regions below the red and yellow dashed lines represent equiaxed grain formation domains for 316L-MCC and 316L alloys, respectively. MCC incorporation induces substantial expansion of the equiaxed grain region. The thermal history during LPBF is obtained by developing a finite element model incorporating heat transfer and convection within the melt pool (fig. S10B). The cooling rate and temperature gradient are extracted from the model, represented by purple data points in Fig. 4B. Because of the ultrahigh temperature gradient (>10^6^ K/m), equiaxed grains are hard to form in 316L steel, while the thermal data predominantly reside within the 316L-MCC equiaxed and mixed zones. This distribution demonstrates MCCs’ efficacy in promoting CET behavior in 316L stainless steel.

According to the above analysis, the microstructural evolution of 316L-MCC samples within the melting pool during LPBF is sketched in Fig. 4C. Upon exposure to the laser beam, the mixed powders undergo rapid melting, and the oxygen and nitrogen absorbed on the powder surface are also dissolved. During cooling, titanium atoms exhibit a preferential affinity for oxygen and nitrogen atoms, leading to the formation of titanium oxynitride above liquidus (*T*_l_). After that, the MCC phase is precipitated immediately following the formation of Ti oxynitride at *T* > *T*_l_. The previously precipitated Ti oxynitride becomes the nucleation site for the MCC phase, and the coarsening of Ti oxynitride is limited by the adsorption of the MCC phase. This interdependent precipitation behavior facilitates the development of core-shell architectures, and they are encapsulated within the cells during rapid solidification. Compared to the low lattice misfit between TiC and ferrite, the doping of Nb and W into TiC can further lower the lattice misfit of MCC shell/Fe interface, thereby facilitating ferrite nucleation (*31*, *32*). The dissolved carbide-forming elements (Ti, W, Nb, Ta, and C) with partition coefficients less than unity are progressively rejected from the solidification front and become enriched in the intercellular liquid regions (*33*). This solute partitioning induces substantial constitutional supercooling ahead of the interface, creating thermodynamically favorable conditions for secondary phase formation. As solidification proceeds, the solute-enriched residual liquid undergoes a eutectic-type reaction, leading to the discontinuous distribution of pure MCCs preferentially along cell boundaries. Hence, compared to the previously formed core-shell particles, these later-formed pure MCCs cannot serve as nucleation sites. After solidification, the equiaxed δ-ferrite grains are transformed into γ-austenite grains through peritectic reaction or direct transformation (*20*).

### Strengthening of material performances

The introduction of MCCs into 316L steels leads to grain refinement, precipitation phase, and dislocation multiplication. The core-shell and pure MCCs are incoherent with the austenitic matrix, thereby contributing to strengthening via the classical Orowan mechanism. Therefore, the total strengthening includes grain refinement strengthening (∆σ_H-P_), Orowan strengthening (∆σ_Orowan_), and dislocation strengthening (∆σ_d_), respectively. The calculated total strengthening (fig. S8D) induced by 4 wt % MCC addition is 428.9 MPa, which is close to the experimental results (384 MPa). The contributions from the above three mechanisms are 113.9, 139.5, and 175.5 MPa, respectively, indicating that the contributions of three strengthening mechanisms induced by the addition of MCCs are comparable. Because of the low number density of pure MCCs (4.3 × 10^12^ m^−2^) compared to that of core-shell MCCs (1.2 × 10^13^ m^−2^), the Orowan strengthening formed by pure MCCs and core-shell MCCs is 36.8 and 102.7 MPa, demonstrating that core-shell precipitation dominates the Orowan strengthening. Moreover, the incorporation of W in the MCC shell structure improves interface wettability, as evidenced by first-principles calculations (*34*). This enhanced interfacial bonding strength facilitates efficient stress transfer while minimizing the typical ductility compromise associated with ceramic reinforcements. Notably, because of ultrafine grains (UFG) (≤2 μm) raising the stacking fault energy (SFE), it is hard to simultaneously achieve the grain refinement and twinning-induced plasticity (TWIP) activation (*20*). However, deformation twins are observed in the 316L-MCC sample, especially near the nano MCC particles (fig. S11). The dissolved Nb and W into the matrix can lower the SFE (*34*, *35*), which can counteract the UFG-induced SFE elevation. Moreover, the high tensile stress (>1 GPa) of 316L-MCC composites may achieve the critical stress of twinning. The activation of deformation twins can enhance the work hardening rate and increase the UE. Therefore, the combined action of these multiscale strengthening mechanisms explains the outstanding strength-ductility balance achieved in MCC-reinforced 316L steels.

**Fig. 5.**
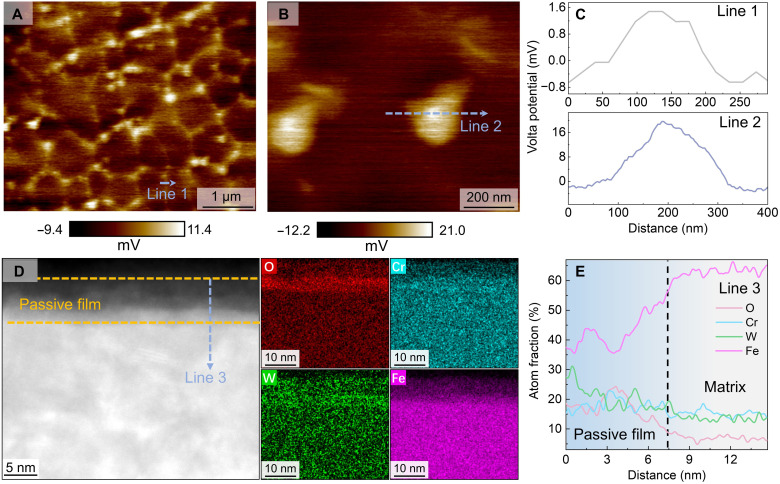
Mechanisms of corrosion resistance in LPBF-fabricated 316L-MCC steels. (**A**) The SKPFM image of cell structure. (**B**) The SKPFM image of MCC/matrix interface. (**C**) Variations of Volta potential along line 1 in (A) and line 2 in (B). (**D**) TEM and EDS images of passive films in 316L-MCC sample immersed in 3.5 wt % NaCl solution for 10 days. (**E**) Concentration profiles along line 3 in (D).

Compared to wrought 316L steels, LPBF-fabricated 316L steels presented superior corrosion resistance in some studies (*36*), whereas others showed inferior properties (*37*). The inconsistency in the corrosion performance usually results from the manufacturing defects (e.g., porosity) and microstructural heterogeneities (*37*). The intrinsic chemical heterogeneity makes Cr- and Mo-depleted interior of cell structures dissolves selectively, possibly accompanied by microgalvanic effect (*36*). However, the incorporation of MCC particles alters this behavior by inducing segregation of W, Nb, and Ta along cell boundaries, thereby modifying Cr distribution. Scheil-Gulliver solidification modeling (fig. S10C) reveals that W, Nb, and Ta segregation reduces the Cr concentration at cell boundaries, narrowing the Cr content difference between boundaries and cell interiors from 6.1 to 4.1%. According to scanning Kelvin probe force microscopy (SKPFM) measurement, the potential difference between cell boundaries and interior is only ~2 mV in the 316L-MCC sample (Fig. 5, A and C), which is lower than previously reported values (4 to 10 mV) in LPBF-fabricated 316L steels (*36*). This confirms that the addition of MCCs reduces Cr segregation and lowers the potential difference, thereby decreasing the risk of selective dissolution and microgalvanic corrosion.

Notably, x-ray photoelectron spectroscopy (XPS) analysis of Fe 2p_3/2_ (fig. S12A) shows a lower Fe^3+^/Fe^2+^ ratio on the 316L-MCC surface, and WO_3_ and W_18_O_49_ are detected in the 316L-MCC passive film immersed in 3.5 wt % NaCl solution for 10 days (fig. S12D). This suggests that dissolved W enhances film stability by forming protective tungsten oxide, suppressing Fe dissolution. The quantitative cationic fraction of 316 and 316L-MCC surface passivation films obtain from XPS results (table S1) also confirms that the doping of W into the passive film enhances the Cr/Fe ratio, which accounts for the thicker and protective inner layer of the passivation film. TEM-EDS analysis of passive film in 316L-MCC sample (Fig. 5, D and E) shows that, a complex and multilayer passive film with a thickness of ~7.5 nm was formed, among which W is mainly rich at the outer layer. This confirms the formation of a stable WO_3_ passivation film, which is consistent with XPS results. The increased interface between the MCCs and the matrix may accelerate microgalvanic corrosion. The SKPFM image of MCC/matrix interface (Fig. 5, B and C) indicates that MCC has a higher potential of ~20 mV than the matrix. The potential difference between MCC and matrix is lower than that of other carbide/matrix interfaces, such as WC/W_2_C (higher by ~85 mV) (*38*) and Cr/Mo carbides (higher by ~100 mV) (*39*), showing the relatively low risk of galvanic coupling. The microstructures of 316L and 316L-MCC samples immersed in 3.5 wt % NaCl solution for 10 days are shown in fig. S12 (E and F). There are elongated pits with a length of several hundred micrometers in 316L steel, which may result from the fact that the pits at different layers connect together (*38*). In comparison, only tiny corrosion pits with a size of several micrometers are found in 316L-MCC samples, proving the superior resistance to pitting corrosion. Therefore, it can be inferred that the corrosion resistance of the 316L-MCC steel is enhanced by multiple mechanisms, including reduced Cr segregation and the formation of a protective passive film of tungsten oxide.

This work demonstrates how MCCs transform additively manufactured 316L stainless steel by co-opting typically detrimental N/O impurities to drive the self-assembly of high-density core-shell nanoparticles [Ti(N,O)-MCC]. These nanoparticles enable an order-of-magnitude grain refinement, unlocking exceptional mechanical strengthening. Simultaneously, the incorporation of W, Nb, and Ta mitigates Cr segregation at cell boundaries while promoting the formation of stable WO_3_ passivation layers, thereby enhancing corrosion resistance. Our findings establish a paradigm for simultaneously controlling grain boundaries and chemical heterogeneity via MCC addition, leading to the synergetic improvement of material strength and corrosion resistance in metal additive manufacturing. This strategy not only repurposes harmful impurities into microstructural assets but also opens a pathway for designing high-performance alloys through targeted nanoparticle engineering.

## MATERIALS AND METHODS

### Sample preparation

Spherical 316L stainless steel powders, produced via gas atomization with an average diameter of 52 μm, were blended with 4 or 6 wt % (TiWNbTa)C MCC particles using mechanical mixing to fabricate the 316L-MCC composite. For a comparison, we also produced 316L-TiC (1 or 4 wt %) composites by the same method. Both MCC and TiC particles size are about 1 μm and fabricated via gas atomization. The uniform distribution of micrometer-sized (TiWNbTa)C particles, identified through Nb and Ta elemental signals, across the surfaces of the predominantly spherical 316L powder particles was confirmed via scanning electron microscopy (SEM) imaging and elemental mapping (fig. S13). Correspondingly, mixed 316L–4 wt % TiC powders are shown in fig. S14. Most TiC particles with micrometer sizes are dispersed evenly on the 316L powder, which is similar to the distribution of MCCs in fig. S13.

Cuboid specimens of 316L, 316L-TiC, and 316L-MCC (10 mm by 45 mm by 10 mm, with the *z* axis as the build direction) were fabricated by a BLT-S210 system (BLT Co. Ltd., Xi’an, China) under the same processing conditions. The process was conducted under an argon atmosphere with oxygen content maintained below 100 parts per million. Key LPBF parameters included a laser scan speed of 950 mm/s, a layer thickness of 20 μm, a hatch spacing of 80 μm, and a 67° interlayer rotation strategy. The chemical compositions of the 316L matrix, MCC particles, and composite are provided in table S2. To evaluate the effects of laser power on microstructure and properties, three power levels, 130, 170, and 210 W, were systematically tested (fig. S15). An optimized laser power of 210 W was used for subsequent experiments. The porosity in 316L and 316L-MCC samples using a laser power of 210 W is 0.003 and 0.008% (fig. S16), respectively, demonstrating that both samples exhibit a high density.

### Microstructure characterization and property testing

Microstructural analyses were conducted using a TESCAN S9000X SEM equipped with electron backscatter diffraction (EBSD) and EDS. Before EBSD measurement, specimens were mechanically polished using SiC papers to 5000 grid and then were electropolished in a solution comprising 20 vol % perchloric acid and 80 vol % ethanol. High-resolution transmission electron microscopy and elemental mapping were performed on an FEI Talos F200X microscope (200 kV) with Super-X EDS. TEM samples were firstly mechanical polished to a thickness of 60 μm, and then a double-jet thinning with an electrolyte composed of 10 vol % perchloric acid and 90 vol % ethanol at 20 V and ~20°C was carried out, followed by foil cleaning via ion milling at a 2° angle and 3 kV for 10 min. Phase identification was carried out via x-ray diffraction (Rigaku D/Max 2550) using Cu-Kα radiation. The continuous scanning mode was used, with a scanning angle ranging from 30° to 100° and a scanning speed of 1°/min. Atom probe tomography (APT) measurements were carried out using a CAMECA LEAP 4000X Si instrument with pulsing ultraviolet laser, an analysis chamber under an ultrahigh vacuum of 3.0 × 10^−11^ torr, at a specimen temperature of 40 K, a pulse laser energy of 50 pJ, a pulse repetition rate of 200 kHz, and a target detection rate of 0.3 to 0.6%. After that, a commercial AP Suite 6.3.3 software was used to reconstruct APT datasets, providing 3D chemical information at near-atomic resolution. For SKPFM characterization, an atomic force microscope (Bruker Dimension FastScan AFM) was used. The specimen was mechanically polished by SiC papers to 5000 grid, followed by electropolishing in a solution comprising 20 vol % perchloric acid and 80 vol % ethanol. The porosity of as-printed specimens (2 mm by 2 mm by 20 mm) was characterized using a Zeiss Xradia 620 Versa microcomputed tomography system with a spatial resolution of 2 μm. 3D reconstruction and data analysis of the acquired images were performed using the Dragonfly software.

Tensile specimens (gauge dimensions: 12 mm by 2.5 mm by 2 mm) were prepared along the *y* direction and tested at ambient temperature on an MTS testing system under quasistatic loading (strain rate: 1.0 × 10^−3^ s^−1^). Strain measurements used a 10-mm-gauge extensometer. Electrochemical performance was assessed via potentiodynamic polarization and EIS using a VersaSTAT MC workstation in neutral 3.5 wt % NaCl solution. Before the experiment, samples with an exposed testing area of 10 mm by 10 mm were mechanically polished using SiC papers to 2000 grid, and then they experienced cleaning, oil removal, and drying to obtain a smooth surface. Polarization scans spanned −0.5 to +1.4 V versus open-circuit potential at 0.5 mV/s, while EIS measurements covered 10^5^ to 10^−2^ Hz with 5-mV perturbation. The steady-final potentials were considered as the change in the potential value of no more than 1 mV in 10 min. Impedance data were modeled using the ZView software. XPS (Thermo Fisher Scientific ESCALAB 250Xi) was used to analyze passive film composition of long-term (10 days) immersion samples, with C 1s peaks referenced to 284.7 eV. XPS spectra were deconvoluted using the MultiPak software.

## Supplementary Materials

### This PDF file includes:

Finite element simulation Strengthening component calculation Figs. S1 to S16 Tables S1 to S3 References
