# Supplementary material for: Strong and corrosion-resistant 3D-printed steel by self-assembled core-shell nanoparticles
Source: Sci Adv. 2026 Jun 10;12(24):eaea5057. doi: 10.1126/sciadv.aea5057 (PMC13251824; doi:10.1126/sciadv.aea5057)
Supplement: Supplementary file 1 — Finite element simulation Strengthening component calculation Figs. S1 to S16 Tables S1 to S3 References [file sciadv.aea5057_sm.pdf]

Science Advances
AAAS

Supplementary Materials for

Strong and corrosion-resistant 3D-printed steel by self-assembled core-shell nanoparticles

Wenhua Wu *et al.*

Corresponding author: Guofeng Zhang, zhanggf@mail.tsinghua.edu.cn;
Youyou Zhang, zhangyyts@mail.tsinghua.edu.cn; Hao Chen, hao.chen@mail.tsinghua.edu.cn

Sci. Adv. **12**, eaea5057 (2026)
DOI: 10.1126/sciadv.aea5057

This PDF file includes:

Finite element simulation
Strengthening component calculation
Figs. S1 to S16
Tables S1 to S3
References

Finite element simulation

To investigate the temperature and convection evolution during laser additive manufacturing, a 3D finite element model was constructed. A Gaussian heat source is set at the top boundary (*58, 59*):

$$Q = \frac{2AP}{\pi R^2} \exp(-\frac{2r^2}{R^2}) \tag{1}$$

where Q is the input heat flux, A is the absorptivity, which is 0.7 at solid phase and is 0.3 at liquid phase. P is the power of laser beam and R is the radial distance in which energy density falls to e^{-2} times that at the center of the laser spot.

The temperature field is calculated using the transient heat transfer equation (*60*):

$$\rho C_p \frac{\partial T}{\partial t} + \rho C_p \boldsymbol{u} \cdot \nabla T = \nabla \cdot (k \nabla T) \tag{2}$$

where ρ is the material density, $\boldsymbol{u}$ is the flow velocity, C_p is the constant pressure heat capacity, T is the temperature, and k is the thermal conductivity.

Flow field is calculated by Navier-Stokes equation (*60*):

$$\frac{\partial \rho}{\partial t} + \nabla \cdot (\rho \mathbf{u}) = 0 \tag{3}$$

$$\rho(\frac{\partial \mathbf{u}}{\partial t} + \mathbf{u} \cdot \nabla \mathbf{u}) = -\nabla p + \nabla[\mu(\nabla \mathbf{u} + \nabla \mathbf{u}^2)] \tag{4}$$

where p is pressure, μ is viscosity, and $\boldsymbol{F_s}$ means volume force that dampens the velocity of the fluid at the liquid-solid phase transition interface and makes the fluid motion in the unmelted powder zone as zero. The modeling details are described in refs. (*58-60*), and calculated parameters are listed in table S2.

Solidification behavior was further analyzed using Scheil-Gulliver simulations in Thermo-Calc 2024. A Gäumann's modified Hunt CET criterion was employed to predict the grain morphology of 316L and 316L-MCC samples, as shown in the following equation (*30*, *61*):

$$\frac{G^n}{R} = a\{\sqrt[3]{\frac{-4\pi N_0}{3\ln(1-\varphi)}} \frac{1}{n}\}^n \tag{5}$$

where G means temperature gradient, R is grain growth rate, N_0 is nuclei density, φ is the volume fraction of the equiaxed grains, a and n are material-dependent constants. The CET diagram shows three sections: fully equiaxed for $\varphi \geq 0.49$, fully columnar for $\varphi \leq 0.006$, and mixed morphology in between.

Strengthening component calculation

The grain refinement strengthening can be calculated by Hall-Petch relationship formula (*40*):

$$\sigma_{\text{H-P}} = 181 + \frac{267}{\sqrt{D}} \tag{6}$$

where D means grain size. The grain size of 316L and 316L-MCC perpendicular to the building direction is 34.1 and 2.8 μm (Fig. 1B), respectively. According to calculations, grain strengthening contributes an increase in yield strength of 113.9 MPa.

The dispersed distribution of MCCs hinders the movement of dislocations by pinning them, leading to Orowan strengthening (*40, 41*):

$$\Delta\sigma_{\text{Orowan}} = \frac{0.538Gb\sqrt{f}}{d} \ln\frac{d}{2b} \tag{7}$$

where G represents the shear modulus and G = 78 GPa for 316L (*40*). b is the Burgers vector (m) and 0.258 nm for 316L (*40*). f represents volume fraction of the MCC particles, and d is particle diameter. The weight percentage of MCCs is 4%, and the average precipitation size is 64.5 nm. The density of 316L and MCC is 8 and ~10 g/cm^3, respectively. Given that nearly all carbon is precipitated according to APT results (Fig. 2E), the volume fraction of particles is estimated to be ~3%. Hence, Orowan strengthening contributes an increase in yield strength of 139.5 MPa. According to the proportion of pure MCCs and core-shell precipitation, Orowan strengthening generated by pure MCCs and core-shell precipitation is 36.8 MPa and 102.7 MPa, indicating that core-shell precipitation dominates the Orowan strengthening.

Considering that the strong crystallographic texture inherent to additively manufactured austenitic stainless steels can significantly affect X-ray diffraction analysis, we employed EBSD-derived geometrically necessary dislocation (GND) density to evaluate the dislocation strengthening contribution. According to fig. S3, the addition of MCC increases GND density (ρ_{d}) from 1.73×10^{14} to 2.53×10^{14} m^{-2}. The increase of yield strength resulted from dislocation density can be obtained by the following equation (*40*):

$$\sigma_{\text{d}} = \beta MGb\sqrt{\rho_d} \tag{8}$$

where β is a constant coefficient and β=1.25 (*40*). M represents the Taylor Factor, which is 3.17 for 316L (*40*). For 316L-MCC, we regard it as randomly oriented FCC polycrystalline material (M≈3.06). The increase of yield strength caused by dislocation density is 175.5 MPa.

The increased yield strength ($\Delta\sigma$) in 316L-MCC compared to 316L can be summarized as: $\Delta\sigma=\Delta\sigma_{H\text{-}P}+\Delta\sigma_{Orowan}+\Delta\sigma_{d}=113.9+139.5+175.5=428.9$ MPa, which is close to the experimental results (384 MPa).

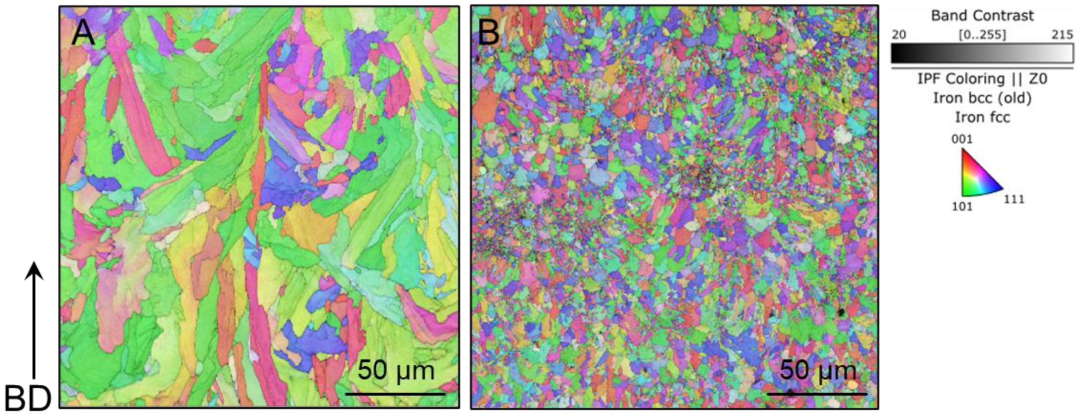

Fig. S1.

Crystallographic characteristics of LPBF-fabricated 316L-TiC with different fractions. (A) Adding 1 wt.% TiC. **(B)** Adding 4 wt.% TiC. The average grain sizes are 25.8 and 5.2 µm, respectively.

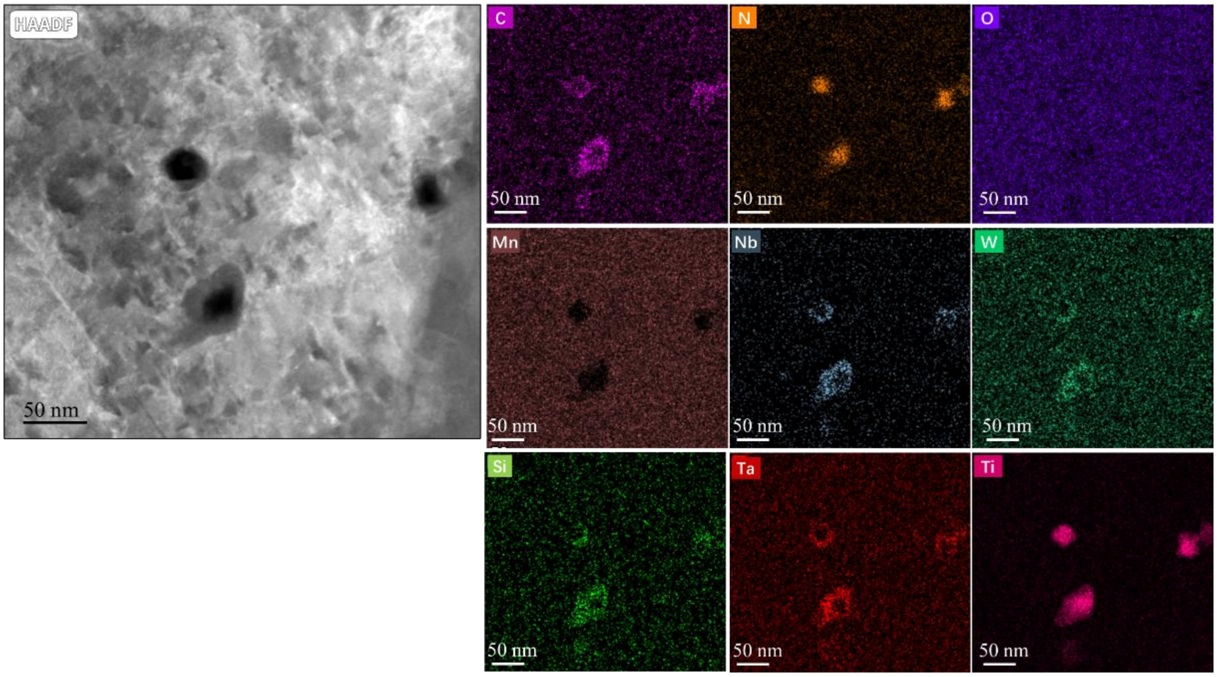

Fig. S2.

TEM-EDS images of 316L-MCC sample.

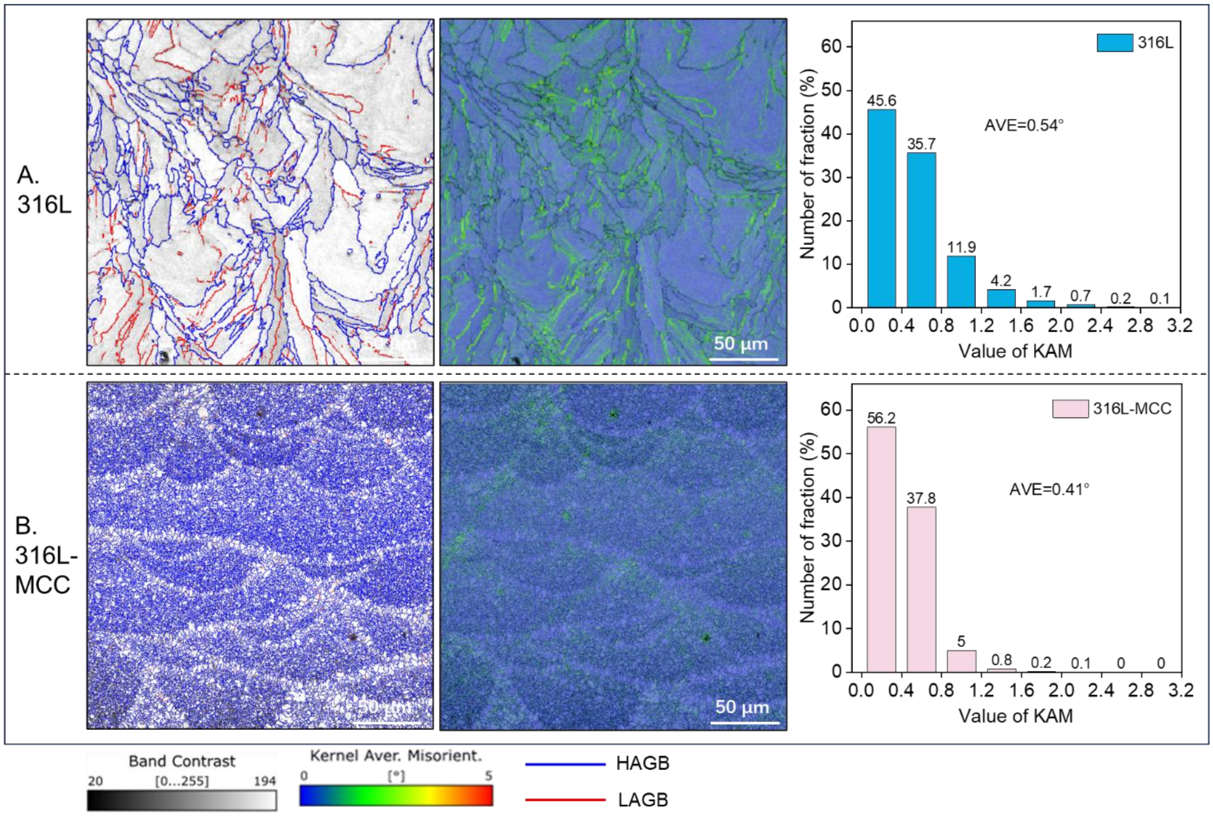

Fig. S3.

Grain boundary and Kernel Average Misorientation (KAM) maps along building direction. **(A)** 316L sample. **(B)** 316L-MCC sample.

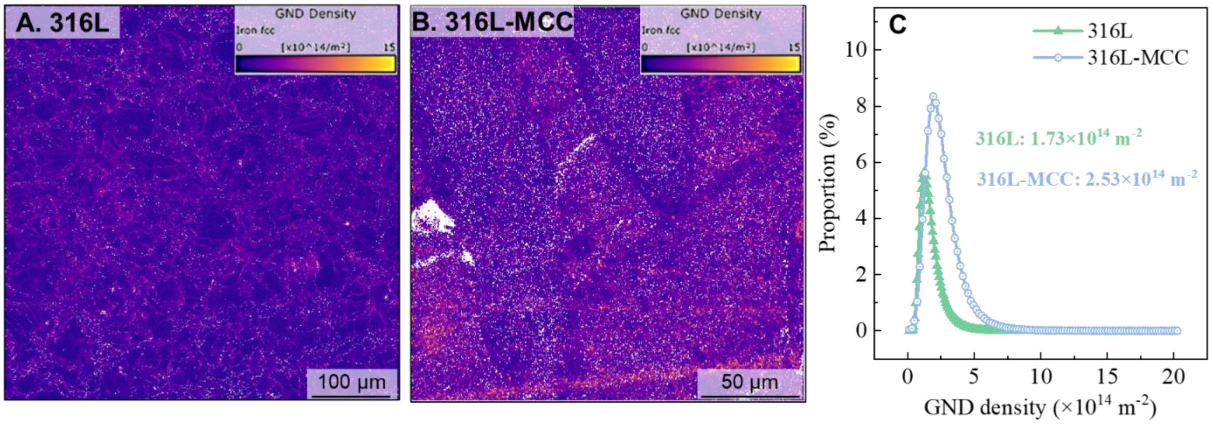

Fig. S4.

GND density distribution of different samples analyzed by EBSD software: (A) 316L sample. **(B)** 316L-MCC sample. **(C)** Statistical distribution map of GND density.

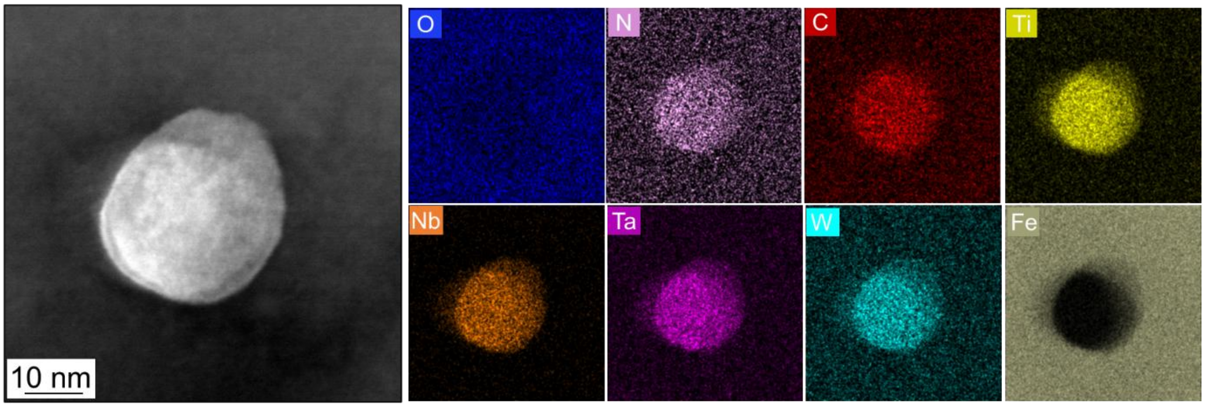

Fig. S5.
TEM and EDS analysis of a pure multi-component carbide (MCC) particle.

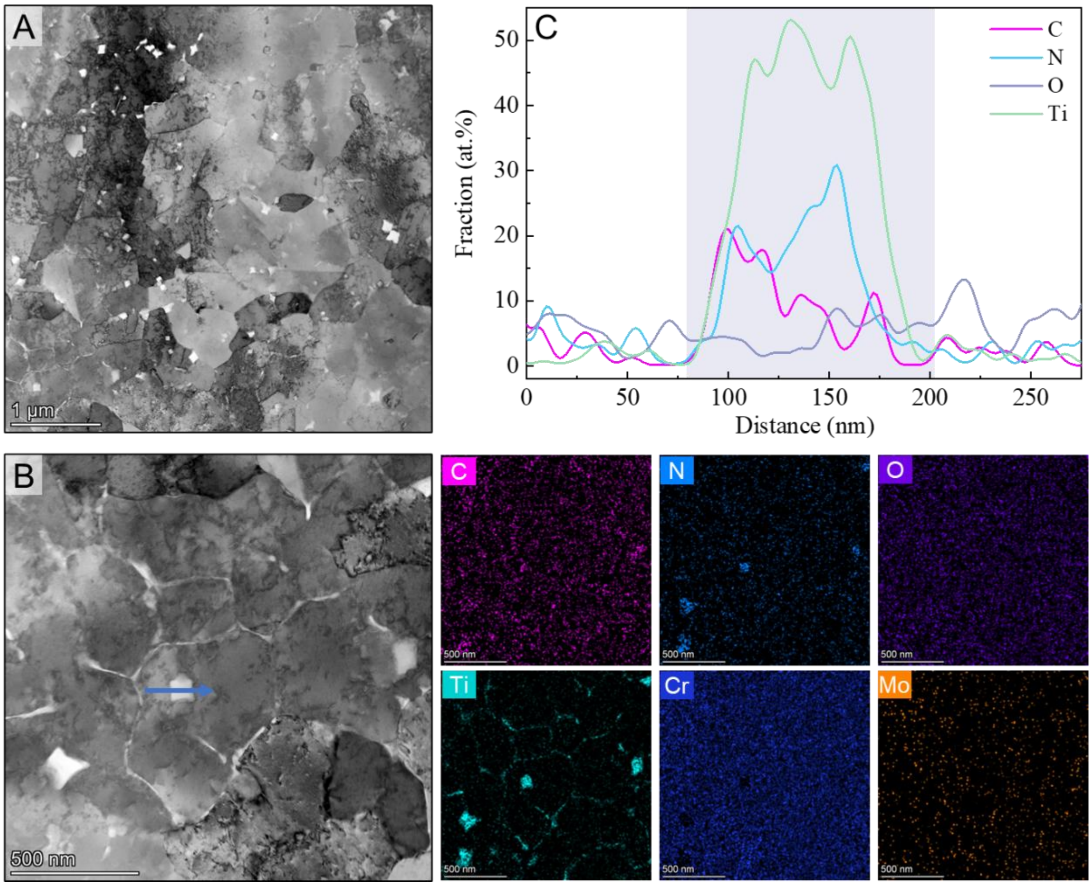

Fig. S6.

Microstructure characteristic of 316L-4 wt.% TiC composite. (A) TEM image showing the distribution of nano-particles on the 316L matrix. **(B)** TEM and EDS analysis of nano-particles. **(C)** Element distribution along blue line in fig. S6B.

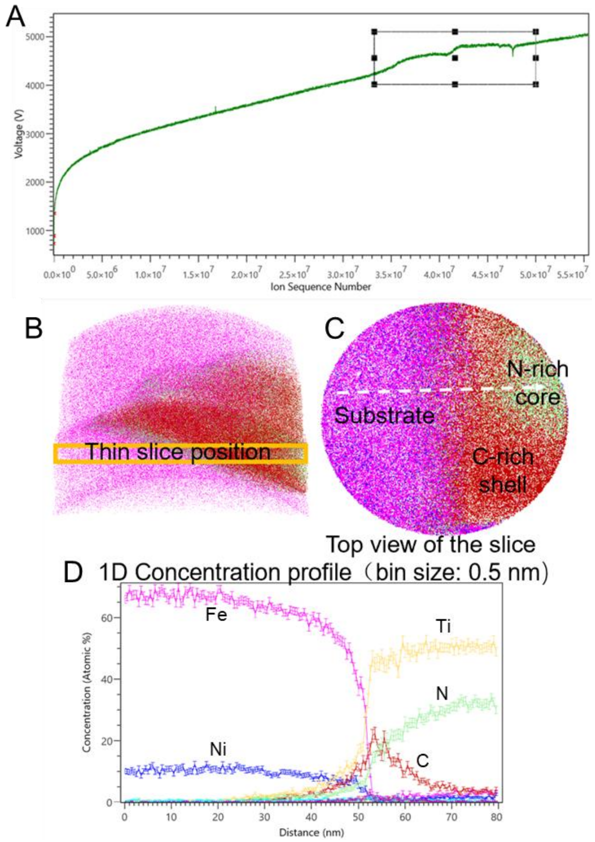

Fig. S7.

APT results of nanoparticles in 316L-MCC steels. (A) Voltage profile of the dataset. **(B)** Combined Fe, C, N atom map of a 3D reconstructed volume with a nanoparticle. **(C)** Top view of a thin slice of the 3D reconstructed volume, showing core-shell structure of the partial nanoparticle with N-rich core and C rich shell. **(D)** 1D concentration profile along the white arrow confirming composition of the nanoparticle.

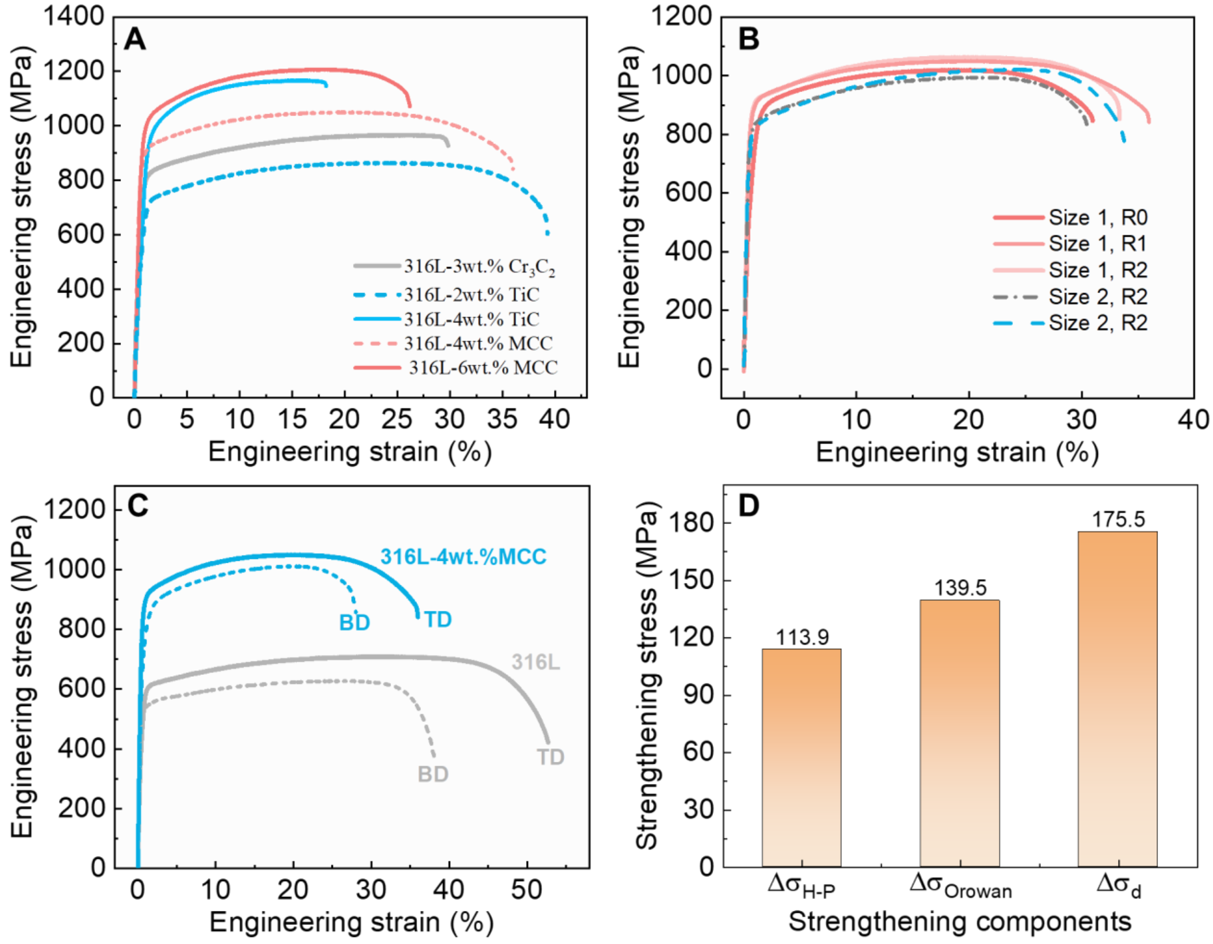

Fig. S8.

Mechanical properties of LPBF-fabricated 316L-based composites. (A) Tensile curves of 316L-based composites under similar printing conditions. **(B)** Tensile curves of 316L-MCC composites under different conditions. Size 1 means the build volume is 10 × 45 × 10 mm^3, and size 2 means that the build volume is 25 × 12 × 15 mm^3. R0, R1 and R2 represent the fresh powder, powder reused once and twice, respectively. **(C)** Tensile curves of 316L steels and 316L-MCC steels along / perpendicular to the building direction. **(D)** Calculated grain refinement strengthening ($\Delta\sigma_{H\text{-}P}$), Orowan strengthening ($\Delta\sigma_{Orowan}$), and dislocation strengthening ($\Delta\sigma_d$) induced by MCCs addition.

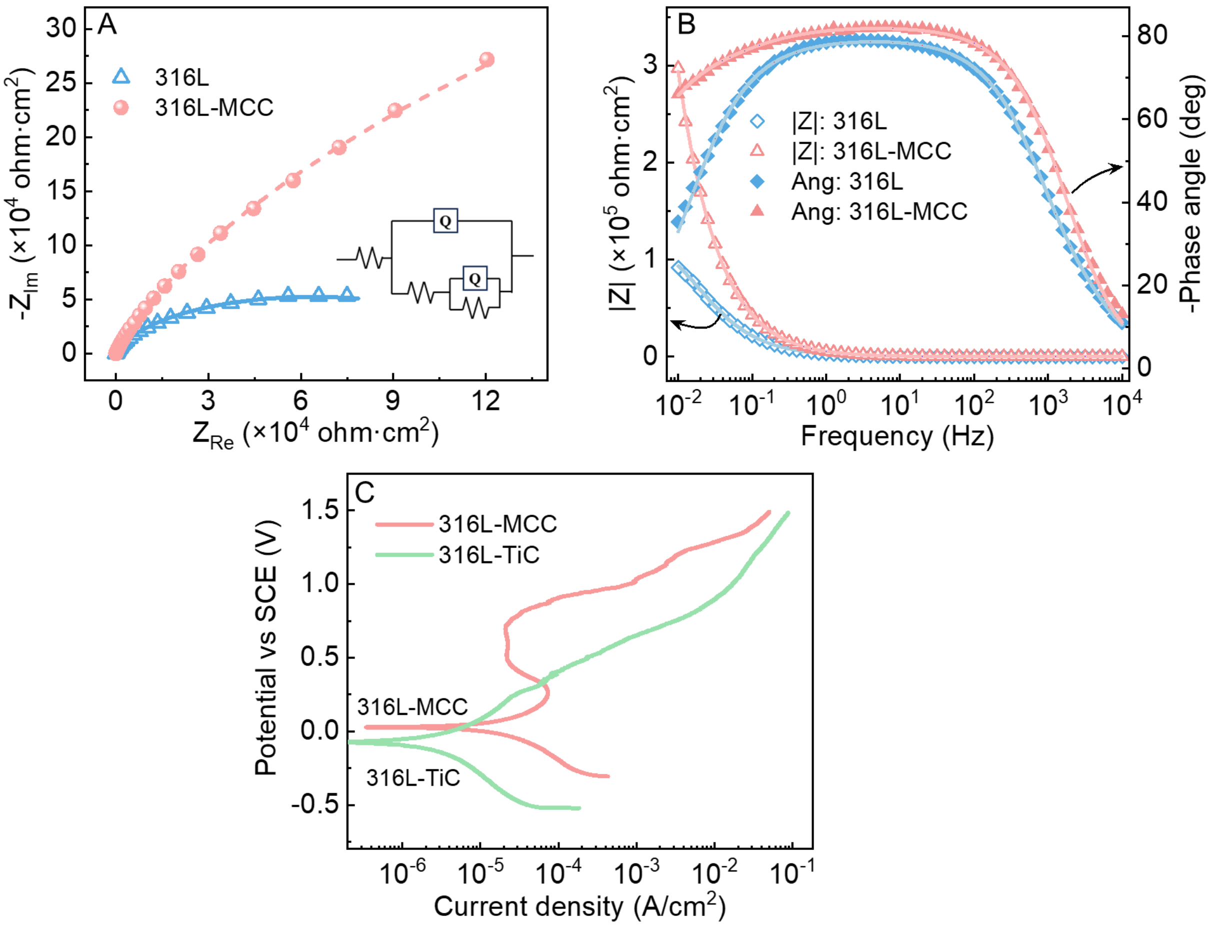

Fig. S9.

Corrosive property of 316L, 316L-1 wt.% TiC and 316L-4 wt.% MCC samples. (A) Nyquist curves and equivalent circuit. **(B**) Bode curves. **(C)** Potentiodynamic curves of 316L-MCC and 316L-TiC steel. A clear passivation range and a higher corrosion potential is formed in the 316L-MCC sample, demonstrating that the 316L-MCC sample exhibits superior corrosion resistance compared to the 316L-TiC sample.

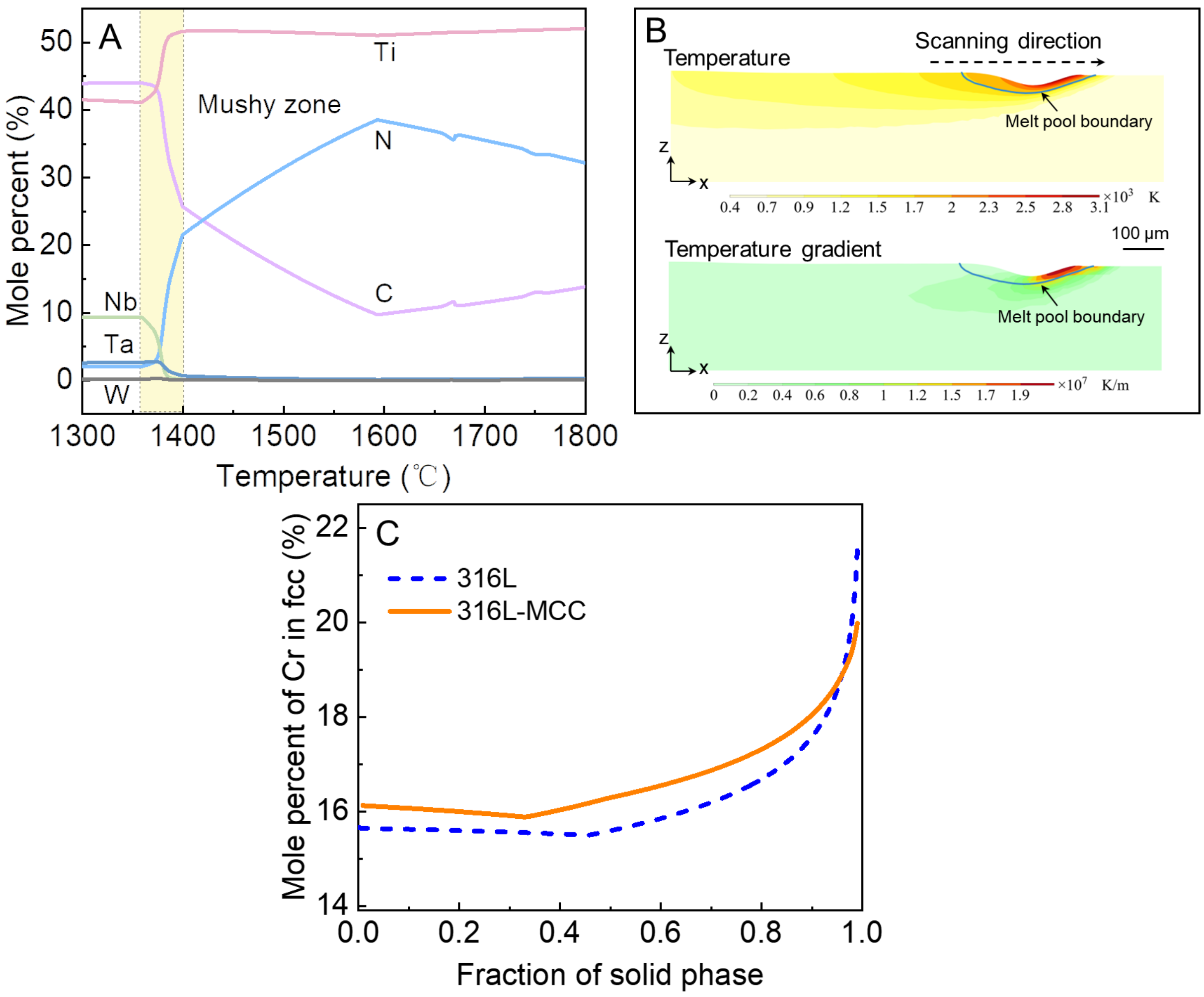

Fig. S10.

Thermodynamic analysis and finite element simulation to reveal the formation of MCCs and thermal history. (A) Element content in MCC phase calculated by Thermo-Calc. **(B)** temperature and temperature gradient along the x-z plane obtained by finite element model. The maximum temperature gradient can reach 10^7 K/m. **(C)** Scheil solidification showing the Cr segregation weakened by the addition of MCCs.

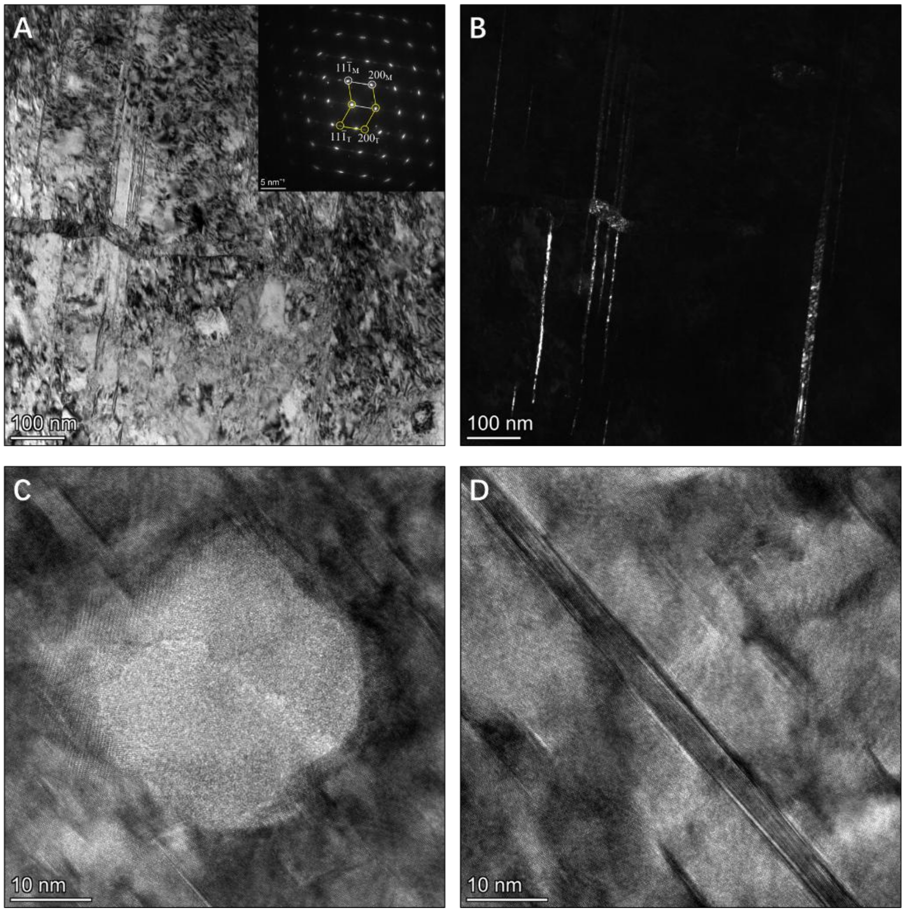

Fig. S11.

TEM analysis of 316L-MCC sample after tensile testing. (A) Bright-field TEM and diffraction spots images showing deformation twins. **(B)** Dark-field TEM image of deformation twins. **(C)** and **(D)** Dislocation pile-ups and deformation twin near a multi-component carbide.

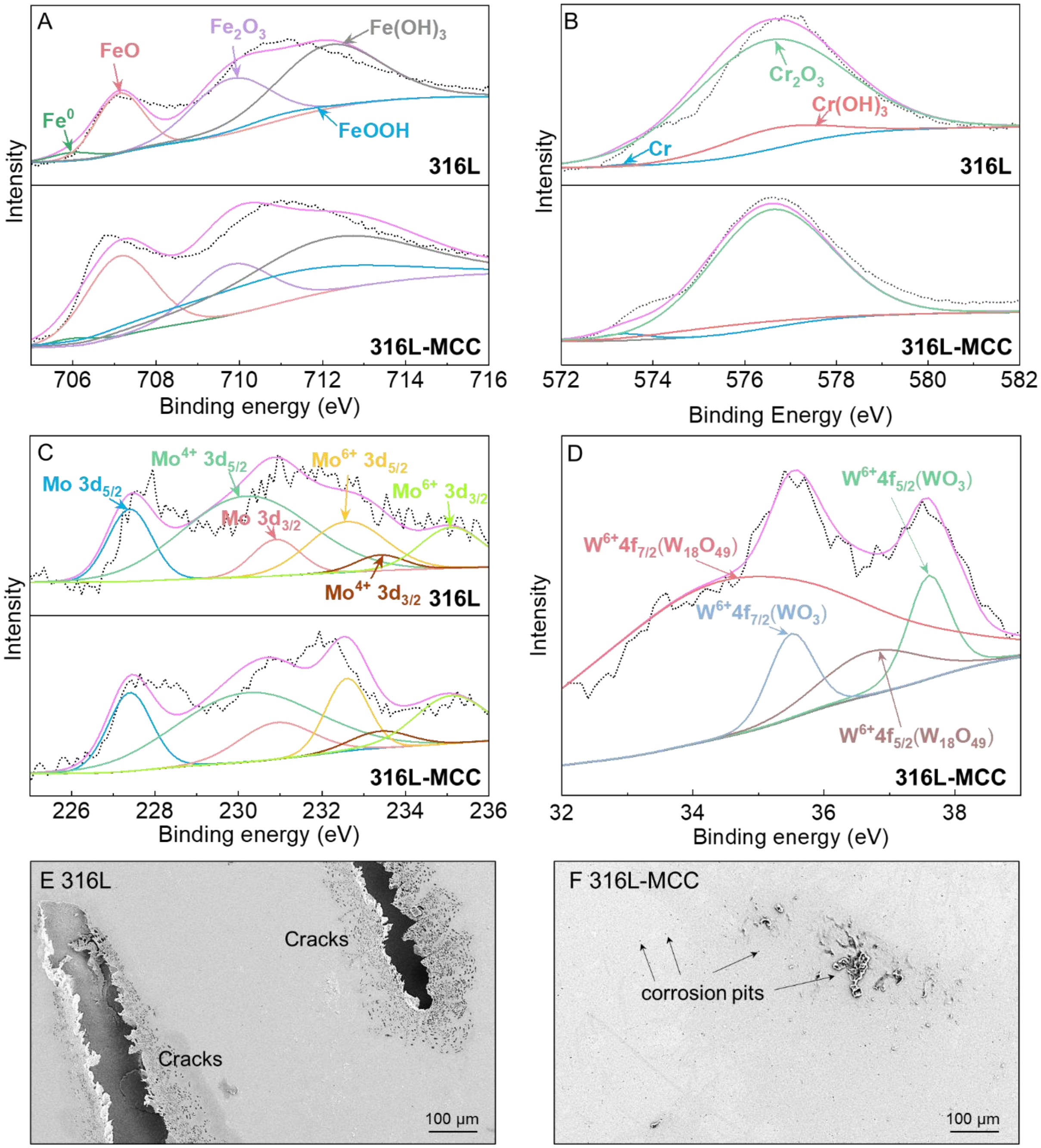

Fig. S12.

Mechanisms of MCCs improving corrosion resistance of 316L steels. XPS results of 316L and 316L-MCC samples immersed in 3.5 wt.% NaCl solution for 10 days: **(A)** Fe. **(B)** Cr. **(C)** Mo. **(D)** W. SEM image of corrosion morphology in **(E)** 316L steel and **(F)** 316L-MCC sample.

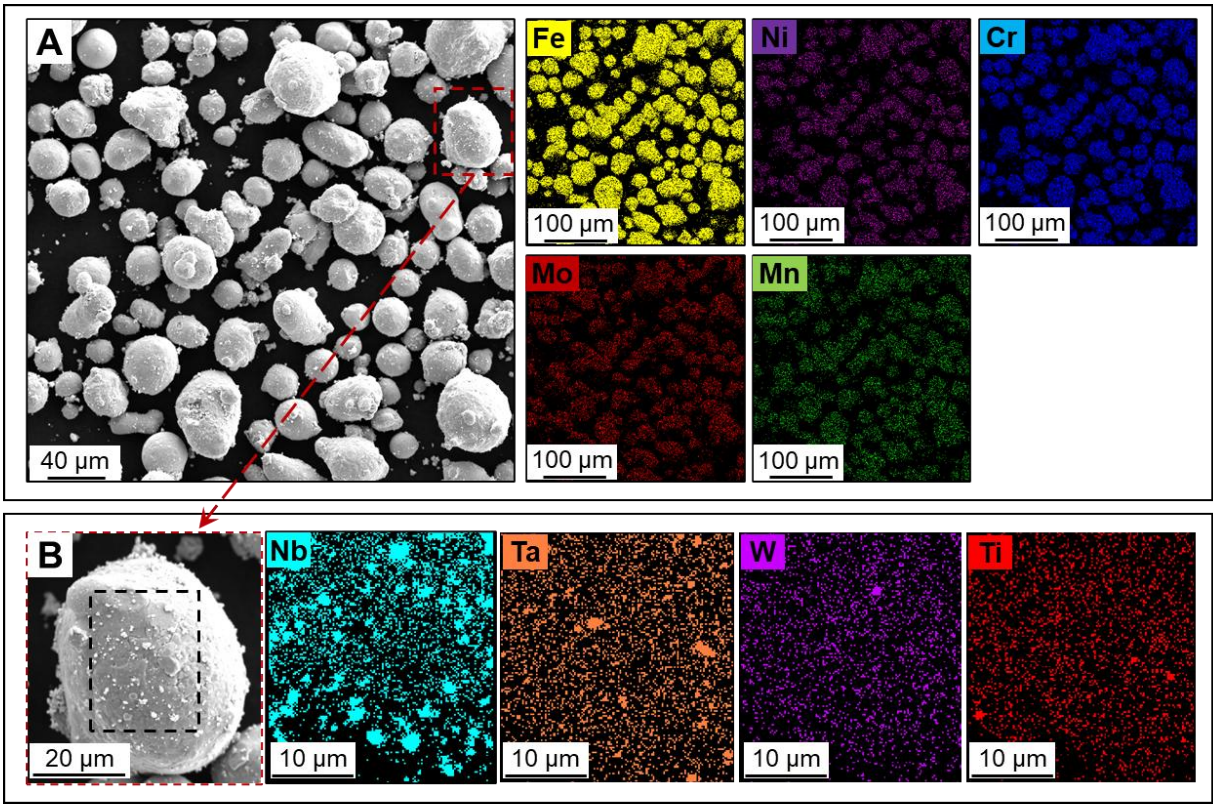

Fig. S13.

SEM image of 316L mixed 4 wt.% MCC particles. (A) 316L-MCC particles and corresponding element map. **(B)** MCC particles distributed at a 316L steel particle and corresponding element map.

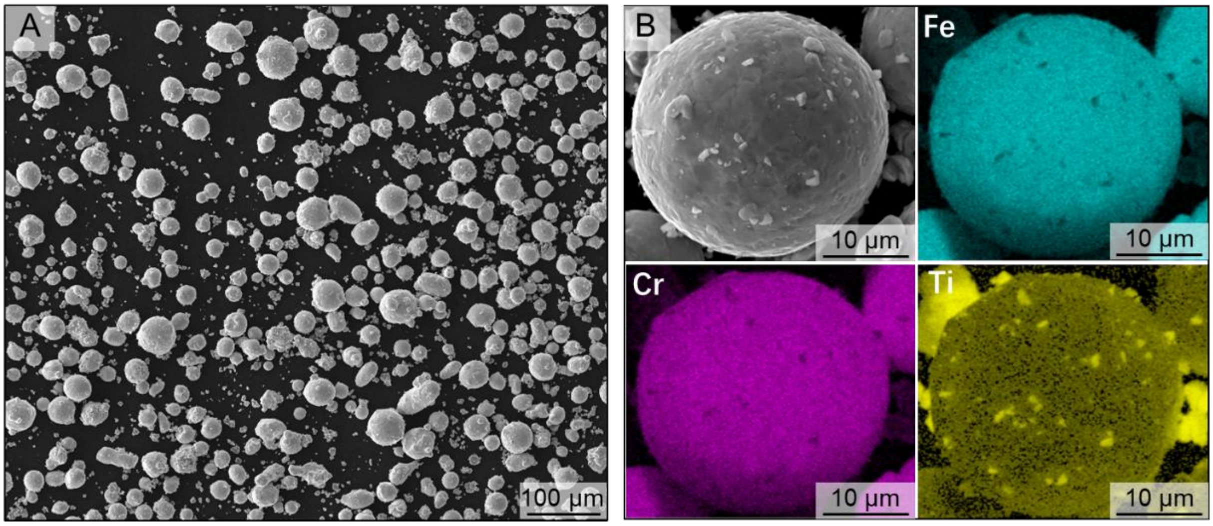

Fig. S14.

SEM image of 316L mixed with 4 wt.% TiC particles. (A) 316L-TiC particles. **(B)** MCC particles distributed at a 316L steel particle and corresponding element map.

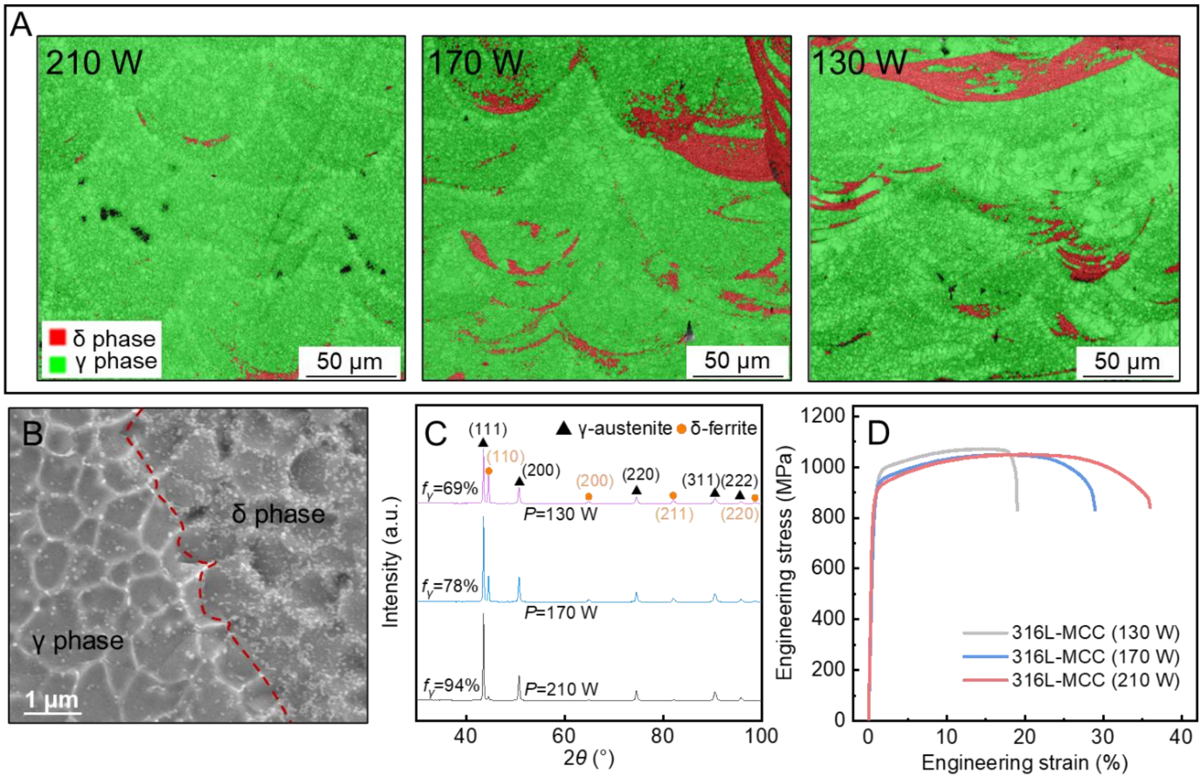

Fig. S15.

The microstructure of the 316L-MCC samples fabricated using various laser power settings. **(A)** EBSD maps of phase distribution in the samples processed at 130, 170 and 210 W. **(B)** A typical SEM image showing many carbides gathering in δ phase when the laser power is 170 W. **(C)** XRD patterns with different laser powers. **(D)** Tensile stress-strain curves of 316L-MCC samples fabricated by different laser powers. The fraction of δ phase increases and is primarily located at the bottom of the melting pool when the laser power is reduced from 210 W to 130 W. Although the fraction of δ phase increases by using lower laser powers, the primary phase is still austenite with the similar grain size, and the MCC content is the same. Therefore, they generate similar grain refinement strengthening, Orowan strengthening, and dislocation strengthening, leading to similar yield strength and initial work hardening rates. However, the increased ferrite-austenite phase boundaries and unfused defects fabricated by low laser power (130 W) are more likely to cause the initiation and propagation of cracks, resulting in the reduction of ductility.

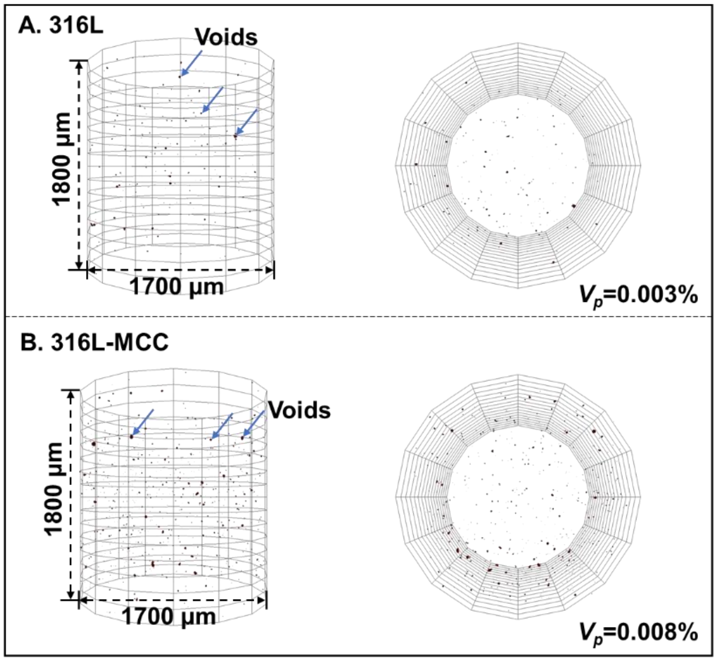

Fig. S16.

3D reconstruction of the micro-CT scans of the as-built samples: (A) 316L steel. **(B)** 316L-MCC composite. V_p means the volume fraction of voids.

Table S1.

The main cationic composition (at%) of the passive films on 316L and 316L-MCC samples obtained by XPS

Specimen	Main cationic fraction in passive film (at%)			
	Fe	Cr	Mo	W
316L	65.16	31.3	3.54	—
316L-MCC	50.53	41.9	3.49	4.08

Table S2.

Chemical content of 316L powders, and 316L, and LPBF-fabricated 316L-4wt.%MCC and 316L-4wt.%TiC bulks (wt.%)

	Cr	Ni	Mo	Mn	Si	Ti	W	Nb	Ta	C	N	O	Fe
316L-powders	16.96	10.95	2.36	0.92	0.77	/	/	/	/	0.02	0.064	0.048	Bal.
316L bulk	16.55	10.74	2.4	0.59	0.6	/	/	/	/	0.006	0.058	0.039	Bal.
316L-MCC bulk	15.62	10.47	2.35	0.55	0.66	0.82	2.12	0.6	0.46	0.18	0.062	0.04	Bal.
316L-TiC bulk	15.59	10.46	2.41	0.54	0.69	3.32	/	/	/	0.78	0.07	0.025	Bal.

Table S3
Parameter used in the finite element model

Parameter	Value	Unit
Liquidus temperature $T_{liquidus}$	1733	K
Solidus temperature $T_{solidus}$	1693	K
Latent heat of phase change L	2.72×10^{5}	J/kg
Viscosity of liquid μ	7×10^{-3}	kg/m/s
Density of alloy	7800	kg/m^3
Constant pressure heat capacity C_p	$330.9+0.563T-(4.015\times10^{-4})T^2+(9.465\times10^{-8})T^3$	J/kg/K
Thermal conductivity k	$11.82+0.0106T$	W/m/K
Mushy zone coefficient C	1.5×10^{5}	kg/m^3/s
Coefficient of surface tension γ_T	-4×10^{-4}	N/m/K
Coefficient of thermal expansion β_T	5.85×10^{-5}	1/K

REFERENCES

1. D. Gu, X. Y. Shi, R. Poprawe, D. L. Bourell, R. Setchi, J. Zhu, Material-structure-performance integrated laser-metal additive manufacturing. *Science* **372**, eabg1487 (2021).

2. Y. Yin, Q. Tan, M. Bermingham, N. Mo, J. Zhang, M. X. Zhang, Laser additive manufacturing of steels. *Int. Mater. Rev.* **67**, 87–573 (2022).

3. T. Mukherjee, J. W. Elmer, H. L. Wei, T. J. Lienert, W. Zhang, S. Kou, T. DebRoy, Control of grain structure, phases, and defects in additive manufacturing of high-performance metallic components. *Prog. Mater. Sci.* **138**, 101153 (2023).

4. B. Guo, D. Cui, Q. Wu, Y. Ma, D. Wei, L. S. R. Kumara, Y. Zhang, C. Xu, Z. Wang, J. Li, X. Lin, J. Wang, X.-L. Wang, F. He, Segregation-dislocation self-organized structures ductilize a work-hardened medium entropy alloy. *Nat. Commun.* **16**, 1475 (2025).

5. Y. M. Wang, T. Voisin, J. T. McKeown, J. Ye, N. P. Calta, Z. Zeng, Y. Zhang, W. Chen, T. T. Roehling, R. T. Ott, M. K. Santala, P. J. Depond, M. J. Matthews, A. V. Hamza, T. Zhu, Additively manufactured hierarchical stainless steels with high strength and ductility. *Nat. Mater.* **17**, 63–71 (2018).

6. O. Gokcekaya, T. Ishimoto, S. Hibino, J. Yasutomi, T. Narushima, T. Nakano, Unique crystallographic texture formation in Inconel 718 by laser powder bed fusion and its effect on mechanical anisotropy. *Acta Mater.* **212**, 116876 (2021).

7. T. Zhang, Z. H. Huang, T. Yang, H. J. Kong, J. H. Luan, A. D. Wang, D. Wang, W. Kuo, C.-T. Liu, In situ design of advanced titanium alloy with concentration modulations by additive manufacturing. *Science* **374**, 478–482 (2021).

8. N. Raghavan, R. Dehoff, S. Pannala, S. Simunovic, M. Kirka, J. Turner, N. Carlson, S. S. Babu, Numerical modeling of heat-transfer and the influence of process parameters on tailoring the grain morphology of IN718 in electron beam additive manufacturing. *Acta Mater.* **112**, 303–314 (2016).

9. Z. Sun, X. P. Tan, M. Descoins, M. Mangelinck, S. B. Tor, C. S. Lim, Revealing hot tearing mechanism for an additively manufactured high-entropy alloy via selective laser melting. *Scr. Mater.* **168**, 129–133 (2019).

10. J. M. Jeon, J. M. Park, J.-H. Yu, J. G. Kim, Y. Seong, S. H. Park, H. S. Kim, Effects of microstructure and internal defects on mechanical anisotropy and asymmetry of selective laser-melted 316L austenitic stainless steel. *Mater. Sci. Eng. A* **763**, 138152 (2019).

11. M. C. Lam, S. C. V. Lim, H. Song, Y. Zhu, X. Wu, A. Huang, Scanning strategy induced cracking and anisotropic weakening in grain texture of additively manufactured superalloys. *Addit. Manuf.* **52**, 102660 (2022).

12. C. J. Todaro, M. A. Easton, D. Qiu, D. Zhang, M. J. Bermingham, E. W. Lui, M. Brandt, D. H. Stjohn, M. Qian, Grain structure control during metal 3D printing by high-intensity ultrasound. *Nat. Commun.* **11**, 142 (2020).

13. D. Y. Zhang, A. Prasad, M. J. Bermingham, C. J. Todaro, M. J. Benoit, M. N. Patel, D. Qiu, D. H. StJohn, M. Qian, M. A. Easton, Grain refinement of alloys in fusion-based additive manufacturing processes. *Metall. Mater. Trans. A* **51**, 4341–4359 (2020).

14. M. Maric, O. Muránsky, I. Karatchevtseva, T. Ungár, J. Hester, A. Studer, N. Scales, G. Ribárik, S. Primig, M. R. Hill, The effect of cold -rolling on the microstructure and corrosion behaviour of 316L alloy in FLiNaK molten salt. *Corros. Sci.* **142**, 133–144 (2018).

15. M. W. Liu, W. Gong, R. X. Zheng, J. Li, Z. Zhang, S. Gao, C. L. Ma, N. Tsuji, Achieving excellent mechanical properties in type 316 stainless steel by tailoring grain size in homogeneously recovered or recrystallized nanostructures. *Acta Mater.* **226**, 117629 (2022).

16. J. H. Martin, B. D. Yahata, J. M. Hundley, J. A. Mayer, T. A. Schaedler, T. M. Pollock, 3D printing of high-strength aluminium alloys. *Nature* **549**, 365–369 (2017).

17. Q. Y. Tan, M. X. Zhang, Recent advances in inoculation treatment for powder-based additive manufacturing of aluminium alloys. *Mater. Sci. Eng. R* **158**, 100773 (2024).

18. D. H. Stjohn, M. Qian, M. A. Easton, P. Cao, The interdependence theory: The relationship between grain formation and nucleant selection. *Acta Mater.* **59**, 4907–4921 (2011).

19. J. Li, H. Q. Qu, J. M. Bai, Grain boundary engineering during the laser powder bed fusion of TiC/316L stainless steel composites: New mechanism for forming TiC - induced special grain boundaries. *Acta Mater.* **226**, 117605 (2022).

20. Q. Tan, H. Chang, G. Lindwall, E. Li, A. Durga, G. Liang, Y. Yin, G. Wang, M.-X. Zhang, Unravelling the roles of TiN-nanoparticle inoculant in additively manufactured 316 stainless steel. *J. Mater. Sci. Technol.* **175**, 153–169 (2024).

21. M. N. Patel, D. Qiu, G. Wang, M. A. Gibson, A. Prasad, D. H. Stjohn, M. A. Easton, Understanding the refinement of grains in laser surface remelted Al-Cu alloys. *Scr. Mater.* **178**, 447–451 (2020).

22. Y. M. Zou, C. L. Tan, Z. G. Qiu, W. Y. Ma, M. Kuang, D. H. Zeng, Additively manufactured SiC-reinforced stainless steel with excellent strength and wear resistance. *Addit. Manuf.* **41**, 101971 (2021).

23. L. Zhang, W. G. Zhai, W. Zhou, X. Q. Chen, L. J. Chen, B. Han, L. C. Cao, G. J. Bi, Improvement of mechanical properties through inhibition of oxidation by adding TiC particles in laser aided additive manufacturing of stainless steel 316L. *Mater. Sci. Eng. A* **853**, 143767 (2022).

24. Z. J. Sun, Y. Xu, F. D. Chen, L. D. Shen, X. B. Tang, L. W. Sun, M. Y. Fan, P. Huang, Effects of ion irradiation on microstructure of 316L stainless steel strengthened by disperse nano TiC through selective laser melting. *Mater. Charact.* **180**, 111420 (2021).

25. M. Ghaffari, A. V. Nemani, S. Shakerin, M. Mohammadi, A. Nasiri, Grain refinement and strengthening of PH 13-8Mo martensitic stainless steel through TiC/TiB2 inoculation during wire arc additive manufacturing. *Materialia* **28**, 101721 (2023).

26. W. Zhai, W. Zhou, S. M. L. Nai, In-situ formation of TiC nanoparticles in selective laser melting of 316L with addition of micronsized TiC particles. *Mater. Sci. Eng. A* **829**, 142179 (2022).

27. V. B. Vukkum, T. Y. Ansell, A. Nieto, R. K. Gupta, Intergranular corrosion of CNT-reinforced and laser powder bed fusion-printed 316L stainless steel. *JOM* **76**, 232–237 (2024).

28. L. F. Liu, Q. Q. Ding, Y. Zhong, J. Zou, J. Wu, Y. L. Chiu, J. X. Li, Z. Zhang, Q. Yu, Z. J. Shen, Dislocation network in additive manufactured steel breaks strength-ductility trade-off. *Mater. Today* **21**, 354–361 (2018).

29. M. L. Qu, J. D. Yuan, A. Nabaa, J. Y. Huang, C. A. Chuang, L. Y. Chen, Melting and solidification dynamics during laser melting of reaction-based metal matrix composites uncovered by in-situ synchrotron x-ray diffraction. *Acta Mater.* **271**, 119875 (2024).

30. M. Gäumann, C. Bezençon, P. Canalis, W. Kurz, Single-crystal laser deposition of superalloys: Processing-microstructure maps. *Acta Mater.* **49**, 1051–1062 (2001).

31. C. Zhao, X. L. Xing, J. Guo, Z. J. Shi, Y. F. Zhou, X. J. Ren, Q. X. Yang, Microstructure and wear resistance of (Nb, Ti) C carbide reinforced Fe matrix coating with different Ti contents and interfacial properties of (Nb, Ti) C/α-Fe. *Appl. Surf. Sci.* **494**, 600–609 (2019).

32. H. Chen, Y. Lu, K. H. Wu, X. Y. Wang, D. L. Liu, Effect of WC addition on TiC reinforced Fe matrix composites produced by laser deposition. *Surf. Coat. Technol.* **434**, 128185 (2022).

33. P. Kumar, S. Huang, D. H. Cook, K. Chen, U. Ramamurty, X. Tan, R. O. Ritchie, A strong fracture-resistant high-entropy alloy with nano-bridged honeycomb microstructure intrinsically toughened by 3D-printing. *Nat. Commun.* **15**, 841 (2024).

34. L. G. Martinez, K. Imakuma, A. F. Padiha, Influence of niobium on stacking.fault energy of all-austenite stainless steels. *Mater. Technol.* **5**, 221–223 (1992).

35. L. Ba, C. Li, J. Pan, Y. Qu, X. Yang, X. Di, Twinning mediated cryogenic toughness in an additively manufactured high Mn steel by manipulating stacking fault energy. *Mater. Today Commun.* **41**, 110424 (2024).

36. E. Delvecchio, T. Liu, Y.-T. Chang, Y. Nie, M. Eslami, A. M. Charpagne, Metastable cellular structures govern localized corrosion damage development in additive manufactured stainless steel. *npj Mater. Degrad.* **8**, 45 (2024).

37. J. Nie, L. Wei, Y. Jiang, Q. Li, H. Luo, Corrosion mechanism of additively manufactured 316 L stainless steel in 3.5 wt % NaCl solution. *Mater. Today Commun.* **26**, 101648 (2021).

38. Y. Q. Zhou, Z. Y. Huang, S. Y. Wang, W. T. Qin, D. C. Kong, T. T. Liu, Y. Yan, X. G. Li, X. H. Qu, D. Engelberg, C. F. Dong, Synergistic improvement of pitting and wear resistance of laser powder bed fusion 420 stainless steel reinforced by size-controlled spherical cast tungsten carbides. *Corros. Sci.* **234**, 112342 (2024).

39. H. Wan, Y. Cai, D. Song, C. Chen, Effect of Cr/Mo carbides on corrosion behaviour of Fe_Mn_C twinning induced plasticity steel. *Corros. Sci.* **167**, 108518 (2020).

40. W. Q. Li, L. X. Meng, S. Wang, H. Y. Zhang, X. F. Niu, H. Lu, Plastic deformation behavior and strengthening mechanism of SLM 316L reinforced by micro-TiC particles. *Mater. Sci. Eng. A* **884**, 145557 (2023).

41. W. Zhai, W. Zhou, S. M. L. Nai, Grain refinement and strengthening of 316L stainless steel through addition of TiC nanoparticles and selective laser melting. *Mater. Sci. Eng. A* **832**, 142460 (2022).

42. X. Meng, J. Yan, B. Ou, Q. He, Y. Zhang, S. Fang, Effect of TaC on microstructure and mechanical properties of 316L stainless steel by selective laser melting. *Mater Charact* **202**, 112990 (2023).

43. F. Lin, L. Liu, S. Yang, NbC particles-reinforced 316L stainless steel fabricated by selective laser melting: Microstructure, mechanical properties, and strengthening mechanisms. *Appl. Phys. A* **130**, 1–18 (2024).

44. D. Chen, Q. Pan, Z. Q. Liu, S. L. Zeng, Q. L. Shi, J. G. Peng, Y. Li, Microstructural evolution, mechanical properties and tribological behavior of SiC reinforced 316L matrix

composites fabricated by additive manufacturing. *J. Mater. Res. Technol.* **30**, 1889–1899 (2024).

45. H. C. Li, Y. B. Hu, R. F. Di, R. W. Yuan, C. Shi, J. B. Lei, Effects of WC particles on microstructure and mechanical properties of 316L steel obtained by laser melting deposition. *Ceram. Int.* **48**, 20388–20399 (2022).

46. Y. Wang, Z. Liu, Y. Zhou, X. Yang, J. Tang, X. Liu, J. Li, G. Le, Microstructure and mechanical properties of TiN particles strengthened 316L steel prepared by laser melting deposition process. *Mater. Sci. Eng. A* **814**, 141220 (2021).

47. C. Ma, M. Grandhi, P. Mallory, Z. Liu, B. Li, B. Kang, Directed energy deposited SS316L with nano-Y2O3 additions: Powder processing, microstructure, and mechanical properties. *Prog. Addit. Manuf.* **10**, 2831–2846 (2024).

48. D. C. Kong, C. F. Dong, X. Q. Ni, L. Zhang, J. Z. Yao, C. Man, X. Q. Cheng, K. Xiao, X. G. Li, Mechanical properties and corrosion behavior of selective laser melted 316L stainless steel after different heat treatment processes. *J. Mater. Sci. Technol.* **35**, 1499–1507 (2019).

49. J. L. Lv, Z. P. Zhou, Z. Q. Wang, Y. D. Xiong, The effect of build orientation on tensile properties and corrosion resistance of 316L stainless steel fabricated by laser powder bed fusion. *J. Manuf. Process.* **106**, 363–369 (2023).

50. M. A. S. Yousif, I. A. Al-Deheish, U. Ali, S. S. Akhtar, K. S. Al-Athel, Mechanical, tribological, and corrosion behavior of laser powder-bed fusion 316L stainless steel parts: Effect of build orientation. *J. Mater. Res. Technol.* **33**, 1220–1233 (2024).

51. A. Zhang, W. P. Wu, M. Wu, Y. X. Liu, Y. Zhang, Q. Q. Wang, Influence of laser power on mechanical properties and pitting corrosion behavior of additively manufactured 316L stainless steel by laser powder bed fusion (L-PBF). *Opt. Laser. Technol.* **176**, 110886 (2024).

52. I. S. Grech, J. H. Sullivan, R. J. Lancaster, J. Plimmer, N. P. Lavery, The optimisation of hot isostatic pressing treatments for enhanced mechanical and corrosion performance of stainless steel 316L produced by laser powder bed fusion. *Addit. Manuf.* **58**, 103072 (2022).

53. Y. Aghayar, A. Shahriari, M. Shakerian, M. Purdy, M. Mohammadi, Microstructure tailoring of laser powder bed fused 316L impellers for enhanced mechanical properties and optimum electrochemical characteristics through hot isostatic pressing. *Mater. Sci. Eng. A* **925**, 147916 (2025).

54. M. Q. Liu, C. R. Jiang, Z. X. Kang, X. Liu, Z. H. Zhang, L. Q. Ren, Effects of graphene addition on the mechanical, friction and corrosion properties of laser powder bed fusion 316L stainless steel. *J. Mater. Res. Technol.* **31**, 170–186 (2024).

55. D. Wang, J. H. Huang, C. L. Tan, W. Y. Ma, Y. M. Zou, Y. Q. Yang, Mechanical and corrosion properties of additively manufactured SiC-reinforced stainless steel. *Mater. Sci. Eng. A* **841**, 143018 (2022).

56. S. I. Ghazanlou, S. I. Ghazanlou, S. I. Ghazanlou, R. Mohammadzadeh, The multifunctional performance of the laser powder bed fusion 316L stainless steel matrix composite reinforced with the CNT-ZrO_2 nanohybrid. *Mater. Chem. Phys.* **313**, 128693 (2024).

57. C. Zhang, J. K. Zhu, C. Y. Ji, Y. Z. Guo, R. Fang, S. W. Mei, S. Liu, Laser powder bed fusion of high-entropy alloy particle-reinforced stainless steel with enhanced strength, ductility, and corrosion resistance. *Mater. Des.* **209**, 109950 (2021).

58. S. Sharma, K. V. M. Krishna, S. S. Joshi, M. Radhakrishnan, S. Palaniappan, S. Dussa, R. Banerjee, N. B. Dahotre, Laser based additive manufacturing of tungsten: Multi-scale thermo-kinetic and thermo-mechanical computational model and experiments. *Acta Mater.* **259**, 119244 (2023).

59. T. Mukherjee, H. L. Wei, A. De, T. Debroy, Heat and fluid flow in additive manufacturing—Part I: Modeling of powder bed fusion. *Comput. Mater. Sci.* **150**, 304–313 (2018).

60. T. Zhang, H. Li, S. Liu, S. N. Shen, H. M. Xie, W. X. Shi, G. Q. Zhang, B. N. Chen, L. W. Xiao, M. Wei, Evolution of molten pool during selective laser melting of Ti–6Al–4V. *J. Phys. D Appl. Phys.* **52**, 055302 (2018).

61. L. Hagen, Z. Yu, A. Clarke, K. Clarke, S. Tate, A. Petrella, J. Klemm-Toole, High deposition rate wire-arc directed energy deposition of 316L and 316LSi: Process exploration and modelling. *Mater. Sci. Eng. A* **880**, 145044 (2023).
